# Multi-targeted metagenetic analysis of the influence of climate and environmental parameters on soil microbial communities along an elevational gradient

**DOI:** 10.1038/srep28257

**Published:** 2016-06-20

**Authors:** Anders Lanzén, Lur Epelde, Fernando Blanco, Iker Martín, Unai Artetxe, Carlos Garbisu

**Affiliations:** 1 Soil Microbial Ecology Group, Department of Conservation of Natural Resources, NEIKER-Tecnalia, c/Berreaga 1, 48160 Derio, Spain; 2 Department of Plant Biology and Ecology, University of the Basque Country (UPV/EHU), 48080 Bilbao, Spain

## Abstract

Mountain elevation gradients are invaluable sites for understanding the effects of climate change on ecosystem function, community structure and distribution. However, relatively little is known about the impact on soil microbial communities, in spite of their importance for the functioning of the soil ecosystem. Previous studies of microbial diversity along elevational gradients were often limited by confounding variables such as vegetation, pH, and nutrients. Here, we utilised a transect in the Pyrenees established to minimise variation in such parameters, to examine prokaryotic, fungal, protist and metazoan communities throughout three consecutive years. We aimed to determine the influences of climate and environmental parameters on soil microbial community structure; as well as on the relationships between those microbial communities. Further, functional diversity of heterotrophic bacteria was determined using Biolog. Prokaryotic and fungal community structure, but not alpha-diversity, correlated significantly with elevation. However, carbon-to-nitrogen ratio and pH appeared to affect prokaryotic and protist communities more strongly. Both community structure and physicochemical parameters varied considerably between years, illustrating the value of long-term monitoring of the dynamic processes controlling the soil ecosystem. Our study also illustrates both the challenges and strengths of using microbial communities as indicators of potential impacts of climate change.

Contemporary climate change is causing changes in species assemblages, often contributing to an accelerating and self-reinforcing loss of biodiversity^1^,^2^. Elevational gradients found in alpine areas are invaluable for studying such changes, as well as their underlying mechanisms and impacts on ecosystem function^3^. The resulting ecotones arranged over short distances, together with their harsh pedoclimatic conditions, also make these alpine habitats particularly sensitive to climate changes and other disturbances. It is therefore not surprising that the effect of contemporary climate change in the Alps (an increase of approximately 2 °C with associated changes in precipitation) has led to changes in vegetation structure and threatened the sustainability of ecosystem services provided^4^,^5^. Microbial communities play fundamental roles for soil ecosystem function and critically influence aboveground communities (and vice versa)^6^. Yet, relatively little is known about such changes in soil microbial communities and their suitability as indicators of climate change impact on soil health.

Examination of elevational gradients in mountain habitats has a long history^3^, but it is only recently that it has been possible to study the composition of their full soil microbial communities, using culture-independent molecular techniques such as fingerprinting and marker gene profiling (“metagenetics”/“metabarcoding”). Although many studies have now used such techniques, no consistent patterns have been found with regard to alpha-diversity. Bryant *et al*. found a monotonic decrease in diversity of soil *Acidobacteria* with elevation along an elevational gradient in the Colorado Rocky Mountains^7^. Similar patterns were observed in total soil bacterial communities at Shennongjia Mountains in Hubei (China)^8^; at Mt. Shegyla (Tibetan Plateau)^9^ and in the Changbai Mountain Range^10^,^11^. Along gradients located on Mt. Fuji and Mt. Norikura (Japan), Singh and colleagues found soil archaeal and bacterial diversity to peak at intermediate elevations and suggested “intermediate disturbance” as a possible explanation^12^. However, the intermediate disturbance hypothesis has been criticised both for being logically incoherent and lacking sufficient empirical evidence^13^. As noted, the observed diversity pattern may also have been caused by a mid-elevation mix of niches and ecotypes, more abundant in the upper or lower zones^12^, or indeed a number of other mechanisms.

Inverse bacterial diversity patterns relative to the above were encountered on Mt. Halla on Jeju Island in the Korea Strait^14^ and in four mountain sites in Yunnan (China)^15^ (i.e. lower diversity at intermediate or lower elevations, respectively). This suggested a lack of universal patterns and that instead a combination of biological and physicochemical factors may influence microbial diversity in a manner that cannot easily be separated from climatic influences^14^,^15^.

Similar to bacterial communities along the same gradient, Shen *et al*. found that the richness of microbial eukaryotes (including fungi and protists) was influenced mainly by soil pH, as opposed to elevation^10^,^16^. Coince *et al*. identified an intermediate peak in fungal rhizosphere OTU richness at intermediate elevations, also noting that *Basidiomycetes* and *Ascomycetes* showed different patterns^17^. These trends were equally explained by elevation and soil pH. Similar mid-domain effects have been observed for the richness of ectomycorrhizal (EM) fungi^18^. General declines in diversity with elevation have also been observed in EM^19^ as well as arbuscular mycorrhizal (AM) fungi^20^.

It is not always clear whether identified correlations between elevation and diversity represent a direct influence of temperature on microbial communities, or other parameters acted as confounding factors. We could only identify one study^21^ where environmental parameters appeared independent of elevation. The mentioned study concluded that no trend in microbial diversity with elevation was present, thus casting doubt on all studies where such patterns were identified^21^. Regardless, in most previous studies^8^,^9^,^12^,^14^,^15^,^16^,^17^,^18^,^21^, soil pH or vegetation structure correlated with alpha diversity or community dissimilarity (“beta diversity”) of soil microbial communities. Total nitrogen and carbon^11^ and exchangeable K^+^ and Ca^2+^ concentrations^15^ in soil have also appeared as main drivers of bacterial communities.

The Ordesa and Monte Perdido Microbial Observatory was established in order to investigate the potential of utilising an elevational gradient for studying how temperature and other aspects of climate may affect soil microbial community structure and function. In order to minimise the effect of confounding factors encountered in several previous studies, a gradient (spanning 1,500 to 2,600 m) was chosen based on the preference of hosting a similar plant community, soil pH, and other physicochemical parameters along the elevational gradient, located in Ordesa and Monte Perdido National Park (Spain), in the Pyrenees. In order to evaluate microbial community structure, we utilised sequencing (Illumina MiSeq) of two sets of amplicons targeting the small-subunit ribosomal RNA of prokaryotes and eukaryotes (16S rRNA and 18S rRNA, respectively), as well as the internal transcribed spacer (ITS) of fungi, and Biolog Ecoplates targeting the functional diversity of heterotrophic bacteria. Here, we aim to characterise, for each of the targeted communities:

– The relative influence of elevation and other abiotic parameters on soil microbial diversity and composition;

– How strongly fungal, protist, metazoan, bacterial and archaeal community structures related to each other;

– How plant community structure related to elevation and soil microbial community structure;

– If a better model explaining community structural changes can be obtained by taking into account temporal variation such as annual and weekly variation, recent soil temperature or snow cover.

This study paves the ground for further characterisation of the effects of climate change on microbial communities, in turn affecting landscape ecology and feedbacks to global carbon and nutrient cycles.

## Materials and Methods

### Study site

The Ordesa and Monte Perdido Microbial Observatory was established in 2011 for the purpose of long-term environmental monitoring, in a protected area surrounding the Valley of Escuaín, in the Spanish side of the Pyrenees mountain range, located inside the Ordesa and Monte Perdido National Park. The area was selected based on its relatively uniform distribution of herbaceous vegetation, orientation and pH along a suitable elevational gradient. For the present study, a transect consisting of twelve sampling stations evenly distributed along an elevational gradient spanning approximately 1,500 to 2,600 m was established on the south and SSW facing slope of the mountain “Tres Marías” (a.k.a. “La Zuca”; see Table 1 and Supplementary Figure S1). The vegetation along the gradient consists of meadows with relatively uniform herbaceous vegetation, thus minimizing a possible source of variation due to vegetation type.

Soil types range from sandy loam to clay with siliclastic or carbonate sedimentary parent rock. Neither parent rock follows any obvious trend related to elevation (Table 1). The area has a long history of pastoralism and the vegetation is affected to some extent by summer grazing cattle and sheep, to roughly the same extent along the gradient. The area is characterised by a typical Pyreneean climate (humid continental to subalpine and alpine) with annual precipitation of approximately 1400 mm at 2000 to 2300 m^22^. The lowest station is close to stands of oak, pine and beech, whereas only a limited amount of wooden species were found above the montane zone (roughly 1500 to 1700 m), likely due to a combination of both grazing and climate (see Table 1). The subalpine zone (1700 to 2300 m) is dominated by the grasses *Festuca eskia* and *F. gauteri*, whereas the alpine zone (2400 to 2600 m) is dominated by *Nardus stricta* (matgrass) and *Poa alpina* (alpine meadow-grass; see Table 1).

### Sample collection and on-site measurements

Soil samples were collected annually between 2011 and 2014 in late August or early September from each station. A number of samples were also taken during other times of the year to study temporal variability (see Table 2). Each sample consisted of a composite of ten soil cores from a plot of 2 × 2 m (10 cm depth, 23 mm diameter). Spatial replicates were taken from such plots separated by more than 2 m. The same sites, adjacent to temperature sensors, were studied each year. Samples were immediately transferred to sterile plastic zip-lock bags, manually mixed in-bag and a subsample taken for molecular analysis, sieved on-site directly after collection (<2 mm) and stored in approximately 10 ml RNALater in 15 ml Falcon tubes. Tubes were mixed and kept at outdoor temperature for a maximum of 48 h and thereafter stored at −20 °C until DNA extraction. No molecular community profiling was carried out during the first year (Table 2).

Slope was measured using the inclinometer of the laser rangefinder TruPulse 360° (Laser Technology Inc., Centennial CO). On-site temperature sensors (HOBO® TidbiT v2, Onset, Cape Cod MA) were planted at each station at a depth of 5 cm during sample collection in August 2012 and resulting measurement data (30 min. intervals continuously throughout the year) retrieved during subsequent sample collections. Due to malfunction of three sensors (Table 2), additional ones were installed 27 October 2013 and data collected during subsequent sample expeditions (with no additional failures). Annual means were calculated based on the daily median, maximum and minimum temperatures for each day in a complete year (26/8 2013–25/8 2014). Similar averages were calculated for the closed 30 day interval previous to each sample collection. The parameter “days under snow cover” was extrapolated by counting the number of occurrences where temperature consistently stayed below 5 °C with under 1 °C daily range of ground temperature (ΔGT) throughout a full (24 h) day. These cut-offs were manually chosen from inspecting annual graphs of ΔGT and mean temperature, since taking into account both can improve accuracy of snow cover prediction^23^.

Plant community characterisation (presence/absence of species) was carried out during the sample collection on 25 August 2013, using three random plots of 0.5 × 0.5 m at each station. Quadrants were thrown randomly from the temperature sensor points. Identification of floral species was challenging due to grazing and being done late in summer, and is to be considered incomplete.

### Soil physicochemical and Biolog Ecoplate measurements

Soils were sieved (2 mm) and air dried at ambient temperature prior to measurements of physicochemical parameters (except humidity). For Biolog Ecoplate analysis, soils were instead stored fresh at 4 °C. Soil humidity was measured by drying at 105 °C for 24 h and re-weighing; and pH measured in deionised water at a soil solution ratio of 1:2.5. Soil particle size distribution (clay, sand and silt) was determined using laser diffraction analysis (Mastersizer 2000, Malvern Instruments, Worcestershire, UK). Soil organic matter (SOM) was measured according to Nelson *et al*.^24^ and total nitrogen (N) according to ISO3878 (1998).

Functional diversity of heterotrophic bacteria was estimated using Biolog EcoPlates (Biolog, Inc., Hayward CA) by counting the number of utilized substrates (NUS) after a 45 h incubation, corresponding to the time of maximal microbial growthfresh at 4 °C for a maximum of two months until analysi^25^.

### DNA extraction and amplicon sequencing

DNA extraction was carried out from 0.25 g soil aliquots from all samples using PowerSoil DNA Isolation kit (Mo-Bio Laboratories, Carlsbad CA), following the manufacturer’s instructions. Prior to DNA extraction, samples were centrifuged (5 min. at 350 × g), the supernatant discarded and the pellet washed with 1× TE buffer and centrifuged again (repeated twice), in order to remove the RNALater solution. Samples were thereafter washed with 20 mM K_2_HPO_4_ to remove extracellular DNA.

Amplification was carried out using a dual indexing tag-tailed design as described by d’Amore *et al*.^26^. Briefly, adapter-linked primers (with N_5_ between forward adapter and primer) were used in the first amplification step using the following reaction to a total of a 20 μl volume: 1 μl template community DNA; 1 μM each of forward and reverse primers; and 1× HotStar PCR mix (QIAGEN, Hilden, Germany). For 18S rRNA amplicons, 1 μl of bovine serum albumin was also added to improve PCR efficiency. The following PCR parameters were used: initial denaturation at 95 °C for 15 min, followed by 25 cycles of 95 °C for 20 s, 55 °C for 30 s, 72 °C for 30 s with a final extension at 72 °C for 7 min. Amplicon libraries were then cleaned using AMPure XP (Beckman Coulter Genomics) and eluted in 25 μl DEPC-treated water. Barcoded primers were used in the second amplification step using the folllowing reaction to a total of 50 μl volume: 5 μl template (cleaned amplicons resulting from amplification step 1), 1 μM each of barcoded forward and reverse primers (adapter-specific), 1× HotStar PCR mix. The same PCR parameters were used as in step 1, except for higher annealing temperature (61 °C), for 10 cycles. Resulting amplicons were visualised on a 1% agarose gel next to products from the first PCR, to verify a unique product and incorporation of barcoded linkers.

The following adapter-linked primer pairs were used: 519F (CAGCMGCCGCGGTAA) adapted from Øvreås *et al*.^27^ and 806R (GGACTACHVGGGTWTCTAAT)^26^, targeting the prokaryotic 16S rRNA hypervariable region V4; 566F and 1200R targeting the eukaryotic 18S region V4^28^; ITS1F (CTTGGTCATTTAGAGGAAGTAA)^29^ and ITS2R (GCTGCGTTCTTCATCGATGC)^29^ targeting the fungal ITS1 region.

Pair-ended sequencing was carried out using an Illumina MiSeq with the V2 kit (approximately 2 × 250 nt length) at the Center for Genomic Research of the University of Liverpool (3 × 16S rRNA, 1 × 18S rRNA and 2xITS sequencing runs) and Tecnalia Corporation, Miñano, Spain (1 × 16S rRNA and 1xITS runs).

### Sequence data analysis and statistics

Read-pairs from 16S rRNA and ITS amplicons were quality-filtered and overlapped using *usearch*^30^ (options *fastq_maxdiff* = *5*, *fastq_maxee* = *0.5*). Overlapped 16S rRNA sequences were then truncated from both ends in order to remove N_5_ and primer sequences (to a length of 252 nt; discarding shorter sequences). Overlapped ITS amplicon sequences were instead trimmed using *cutadapt*^31^ to remove the reverse primer, because of their variable length. 18S rRNA reads could not be overlapped due to the larger amplicon length (>600 nt) and therefore reverse reads were discarded, while forward reads were otherwise quality filtered and cropped as described above (*fastq_maxee* = 0.5) to a length of 221 nt after removing forward linker and primer sequences. Reads not matching the forward primer exactly were discarded.

All quality-filtered overlapped sequences from 16S rRNA, 18S rRNA and ITS amplicons, respectively, were merged across datasets and clustered into OTUs at 97% sequence similarity using *vsearch*^32^. Clustering included de-replication, sorting by abundance (descending and not retaining singletons), then clustering into OTUs at 97% sequence similarity and finally chimera filtering using the *uchime* reference-based followed by the *uchime de novo* method. As reference databases for chimera filtering, we used the ChimeraSlayer *gold.fa* (for 16S rRNA), UNITE^33^ (for ITS) and SilvaMod v106 (for 18S rRNA)^34^.

Representative OTU sequences were aligned to the SilvaMod v106 (16S and 18S rRNA) and UNITE (ITS) reference databases using *blastn* (v.2.2.25 + task megablast) and taxonomically classified using CREST using default parameters^34^. Resulting taxon distributions were studied at order rank as determined by CREST. 18S rRNA taxon data was also divided further into fungi, metazoa and protists (all eukaryotic taxa considered unicellular, except for metazoa or fungi). Relative OTU and taxon abundances were used in further analysis (sum of read abundances mapped to an OTU, or taxon including child nodes, in a particular sample classified, divided by total sample reads). This approach was motivated by the fact that sequence-based relative abundance has been demonstrated to provide meaningful semi-quantitative information when comparing community structure between samples^35^, in spite of being affected by issues such as ribosomal copy number variability and preferential amplification.

Multivariate statistics, calculation of rarefied richness and visualization was performed using the R package *vegan*^36^. Rarefied richness estimates interpolating the expected richness at the lowest sample-specific sequencing depth were used to compensate for variation in read numbers across samples. OTU distributions were transformed into relative abundances using the function *decostand.* These were subjected to Hellinger transformation before calculation of Bray-Curtis dissimilarity matrices comparing community composition between samples. Non-metric multidimensional scaling (NMDS) using function *metaMDS* and Mantel-tests were performed using these dissimilarity matrices. The later was used to compare dissimilarity matrices between the different communities/markers (e.g. fungal ITS-based to prokaryotic 16S rRNA-based), limited to the subset of samples for each such pairwise comparison where sequence data existed for both markers.

Continuous variables were fitted to the resulting NMDS space using the function *envfit.* For this purpose, parameters were divided into groups based on availability of data, since certain measurements were only available for a subset of samples, and each such group compared independently to the NMDS. The function *bioenv* in *vegan* was used to find the subset of parameters that together showed maximum correlation with community dissimilarity. This and subsequent analysis was limited to samples where such parameter measurements were available. Multivariate analysis of variance (MANOVA) and multiple regression on dissimilarity matrices (MRM) models were assessed based on this variable selection, as implemented in the *vegan* function *adonis* and the function *MRM* of the *ecodist*^37^ package, respectively. ANOSIM (analysis of similarity) was carried out based on Bray-Curtis dissimilarities in order to evaluate the effect of factors (sampling year, orientation and parent rock) on community structure.

Correlation analyses were carried out in order to compare: (1) all soil physicochemical and climate-related parameters to elevation; (2) diversity data (including relative shares of eukaryotic groups, and NUS) with elevation; (3) diversity data to soil physicochemical and climate-related parameters; (4) different diversity estimates to each other; and (5) relative taxon abundances with elevation, soil physicochemical and climate-related parameters. Correlations between continuous variables were determined using Kendall’s rank correlation; and between continuous variables and factors using group-wise ANOVA and Tukey’s Range Test (Honest Significant Difference). Coefficients of determination (adjusted R^2^) were determined using linear regression. All correlation analyses were subjected to Bonferroni correction and not reported unless p < 0.05 after correction.

## Results

### Temperature and physicochemical parameters

**Figure 1:**
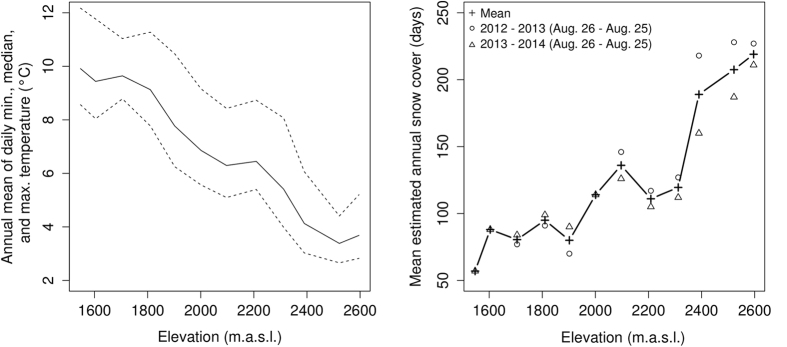
Annual mean of daily minimum, median and maximum soil temperatures (2013–2014), and predicted days under snow coverage, along the studied gradient.

Soil temperature during sampling (T_0_) ranged from 8 to 26 °C; annual means of daily median soil temperature ranged from 3 to 10 °C, and estimated annual snow coverage from 57 to 219 days, along the studied gradient (Fig. 1, Supplementary Table S1). These variables correlated significantly to elevation, as expected (Table 3), although not monotonically (see Fig. 1), and less strongly for T_0_ as expected given the inclusion of samples from other seasons. Temperature variation throughout the year was relatively consistent between the two years measured, with strong variations in daily minima, medians and maxima, especially during spring and summer (Supplementary Figure S2).

Soil humidity (ranging between 11–24%), pH (4.5–7.7) or carbon-to-nitrogen ratio (C/N; ranging between 7–19) showed no significant correlation with elevation (Table S1); however, sites with carbonate parent rock harboured soils with significantly higher pH, as expected (p < 10^−15^). On the other hand, soil texture correlated significantly with elevation, as more elevated sites tended to contain more sand and less clay (Table 3, Supplementary Figure S3). Supplementary Table S2 lists all correlations between measured parameters. Climatic parameters (annual soil temperature averages and predicted days of snow) did not correlate significantly with any other parameters apart from those correlating with elevation.

### Influence of climate and environmental parameters on microbial alpha-diversity

Amplicon sequencing resulted in over 10 M prokaryotic 16S rRNA reads clustered into 22,231 OTUs (n = 60 samples; mean sequence length 252 bp), 5 M eukaryotic 18S rRNA reads clustered into 8,957 OTUs (n = 39; mean length 253 bp), and 9 M fungal ITS reads clustered into 6,035 OTUs (n = 37; mean length 221 bp), after quality filtering and removal of singletons (Supplementary Table S3). Only rarefied (interpolated) OTU richness was considered further, in order to compensate for differences in sequencing depth (number of reads) between samples. Rarefied richness estimates varied between 2275–5260 for prokaryotes, 704–2767 for eukaryotes and 310–1102 for fungi (Table S3. No correlation between elevation and rarefied richness could be identified.

**Figure 2:**
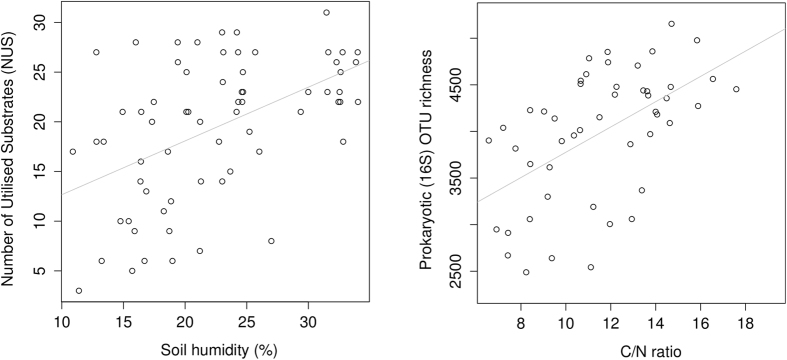
Identified correlations between alpha-diversity estimates and physicochemical parameters.

As opposed to elevation, two diversity estimates correlated significantly with soil physicochemical parameters (see Table 3): Number of Utilised Substrates (NUS) with soil humidity, and prokaryotic rarefied richness with C/N (Fig. 2).

### Influence of climate and physicochemical parameters on soil microbial community composition

A linear regression between vertical distance and 16S rRNA-based Bray-Curtis dissimilarity indicated that prokaryotic communities (“beta-diversity”) was structured by elevation (p = 2 × 10^−5^, see Supplementary Figure S4). The correlation between vertical distance and difference in annual average of daily maximum soil temperature was stronger, indicating that the elevational pattern could likely be explained by climatic differences (Supplementary Figure S4). The relationship between community dissimilarity and different climatic and physicochemical soil parameters was further investigated using multivariate statistics.

**Figure 3:**
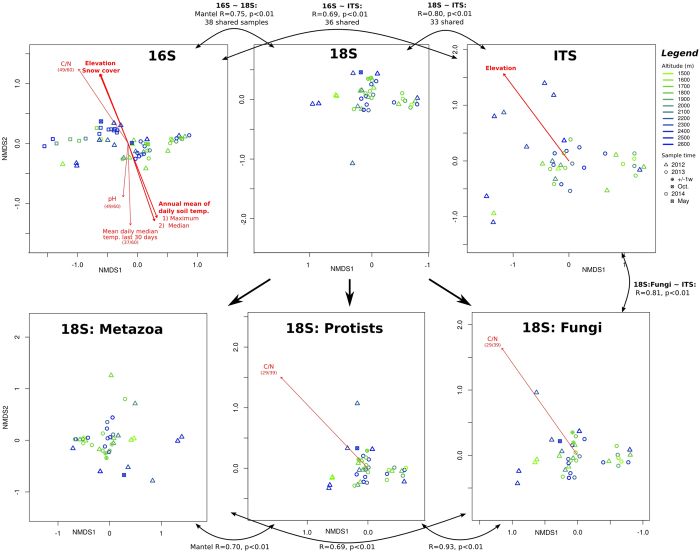
Non-metric multidimensional scaling (NMDS) based on Bray-Curtis dissimilarities of microbial community composition. Composition was based on Hellinger-transformed relative OTU abundances from prokaryotic 16S, eukaryotic 18S and fungal ITS amplicon data. 18S data was also divided into organism groups. Sites are labelled according to legend and red vectors indicate fitted environmental parameters significantly correlated to NMDS coordinates. Where parameter measurements were only available for a subset of samples, this is indicated in parenthesis (samples/total), whereas thick lines indicate that measurements were available for the complete dataset. Black bidirectional arrows illustrate Mantel tests for similarity between community dissimilarity matrices.

NMDS based on compositional dissimilarity between samples did not result in any clear clustering pattern in relation to elevation or year for any dataset (Fig. 3). However, fitting of environmental parameters to the resulting NMDS indicated that elevation correlated with both prokaryotic and fungal community dissimilarities (the later only significant for the ITS dataset, see Fig. 3).

C/N ratio showed the strongest correlation with prokaryotic OTU composition, and also correlated with protist and fungal NMDS coordinates (based on 18S rRNA amplicon subsets; Fig. 3 and Supplementary Table S4). Prokaryotic composition also correlated to soil pH, annual soil temperature averages (based both on daily medians and maxima), predicted days of snow coverage, and recent soil median temperature 1–30 days prior to sampling (Fig. 3). BIOENV analysis, excluding the incomplete data of recent temperature data, indicated that prokaryotic community dissimilarity was best explained by a combination of C/N ratio, pH and annual average of daily maximum soil temperature. A MANOVA/*adonis* model, as well as MRM, verified that each of these parameters contributed significantly to explain community dissimilarity, together accounting for 28% or 12% of variation, respectively (see Table 4). Modifying these models to use elevation or daily median instead of maximum temperature consistently resulted in slightly larger residuals and lower significance. No corresponding, significant models of fungal or total eukaryotic communities could be obtained.

ANOSIM indicated that prokaryotic composition also differed significantly depending on sampling year, parent rock and orientation; whereas eukaryotic composition differed between years (Table 5). Results from protist, fungal and metazoan subsets were essentially identical to total eukaryotes (data not shown).

### Taxonomic composition

We chose to study taxonomic distribution at order rank where loss of information regarding rarer OTUs could be compensated by a relatively higher taxonomic resolution for more abundant OTUs. Out of the quality-filtered reads, 91, 76 and 68% could be taxonomically classified to this rank for 16S rRNA, 18S rRNA, and ITS amplicons, respectively, representing 73, 28 and 33% of unique OTUs. Based on classification at phylum rank (96% of reads), 18S rRNA data was further subdivided into Metazoa, Fungi and “protists” (non-fungal taxa dominated by unicellular organisms). Fungi dominated most samples (contributing to 76% of total reads), followed by protists (10%) and Metazoa (7%; see Table S3). However, protist communities appeared most diverse with 2,729 OTUs.

**Figure 4:**
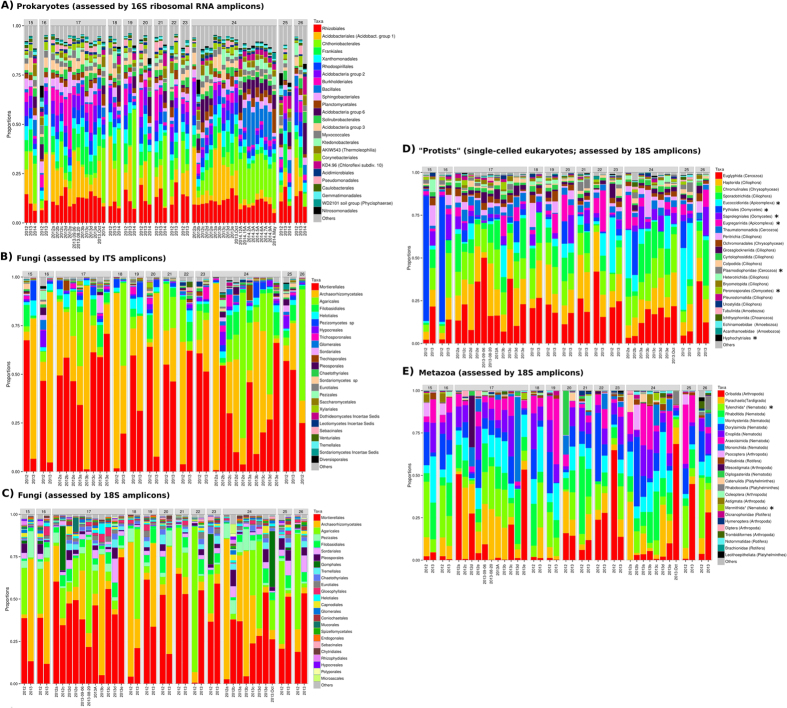
Distribution of most abundant taxa across samples. Relative abundances of taxa at order level are presented as bar-charts for each individual sample, grouped by elevation (in 100 m) for the three specific amplicon library types prepared. Total 18S amplicon results are divided by organism type into fungi, “protists” and metazoa. Typically parasitic taxa are marked with asterisks.

Figure 4 illustrates the relative abundances of the most abundant taxa at order rank for each taxonomic subset (prokaryotes, protists and metazoa) or amplicon-specific subset (fungi as assessed using ITS and 18S rRNA). Prokaryotic communities appeared less heterogeneous compared to eukaryotes. Nonetheless, there was a relatively large variability in dominating bacterial taxa. The most common taxa were Rhizobiales, followed by Acidobacteriales, Chthoniobacterales (of the Verrucomicrobia) and Frankiales (Fig. 4A). Archaea contributed only 0.14% of prokaryotic abundance.

ITS and 18S amplicon data provided very similar taxonomic profiles of the fungal communities (Fig. 4B,C). Both datasets agreed about fungal communities being dominated by Mortierellales, followed by Archaeorhizomycetales and Agaricales. Together, these three taxa contributed between 60–95% of relative abundance, but their individual abundances varied strongly between sites and spatial or temporal replicates.

Four taxa together dominated the protist communities in most samples (Fig. 4D): Euglyphida (filose amoebae of the Cercozoa), Haptorida (ciliates), Chromulinales (flagellates of the Chrysophycae), and Sporadotrichida (ciliates). Taxa with a typical parasitic lifestyle belonging to the Apicomplexa or Oomycetes were also common (marked in Fig. 4D) and some samples were dominated by the Pythiales or Eucoccidiorida. All reads from the former taxon could be classified as *Pythium monospermum*–a saprotroph capable of opportunistic infection of free-living nematodes^38^. Apicomplexans could be classified at family level to Cryptosporidiidae and Monocystidae, constituting roughly one and two thirds, respectively, of apicomplexan abundance. The former are well-known parasites of Annelida while the later are typically associated with vertebrate hosts.

The most abundant metazoan taxa were Oribatida (moss mites), the Eutardigrade order Parachaela and the parasitic nematode order Tylenchida (Fig. 4E). Several other orders of nematodes and arthropods were also identified, as well as rotifers and Platyhelminthes (flatworms). The majority of Oribatida sequence reads were classified as *Tectocepheus sarekensis*.

No correlation between abundances of individual taxa and elevation could be identified, but 44 taxa correlated significantly with other parameters (Supplementary Table S5). All taxa except Group I.1c Thaumarchaeota were bacterial, corresponding to as many as 25% of all bacterial taxa. Of these, most (n = 41) correlated with C/N, notably Rhizobiales and Acidobacteriales (both negatively). Group I.1c abundance also correlated with C/N, negatively (Table S5). Two taxa correlated with pH (Corynebacteriales and Chloroflexi Subdivision 10: P2-11E) and one with slope (Desulfuromonadales).

### Correlation of bacterial, archaeal, fungal, protist and metazoan communities

Mantel tests comparing Bray-Curtis dissimilarity matrices from different amplicon types revealed strong correlation between the prokaryotic, total eukaryotic and fungal community composition (0.69 ≤ R ≤ 0.75; p < 10^−3^; see Fig. 3). Fungal community profiles using 18S rRNA vs. ITS correlated yet more strongly (R = 0.81). Comparing the three eukaryotic organism groups revealed that fungal and protist communities were most correlated (R = 0.93; Fig. 3). Prokaryotic, total eukaryotic and fungal rarefied richness estimates also correlated significantly (Table 3). Further, samples with a higher relative abundance of protists compared to total eukaryotes appeared to harbour more diverse prokaryotic as well as fungal communities, whereas fungal abundance appeared negatively correlated with fungal richness (Table 3). Comparing rarefied richness of fungi between 18S rRNA and ITS amplicon datasets revealed strong correlation between the two approaches, with 18S rRNA-based estimates being significantly lower (n = 31, slope 0.57 ± 0.05, intercept 140 ± 27, p < 10^−12^; see Table 3 and Supplementary Figure S5).

### Plant community structure

**Figure 5:**
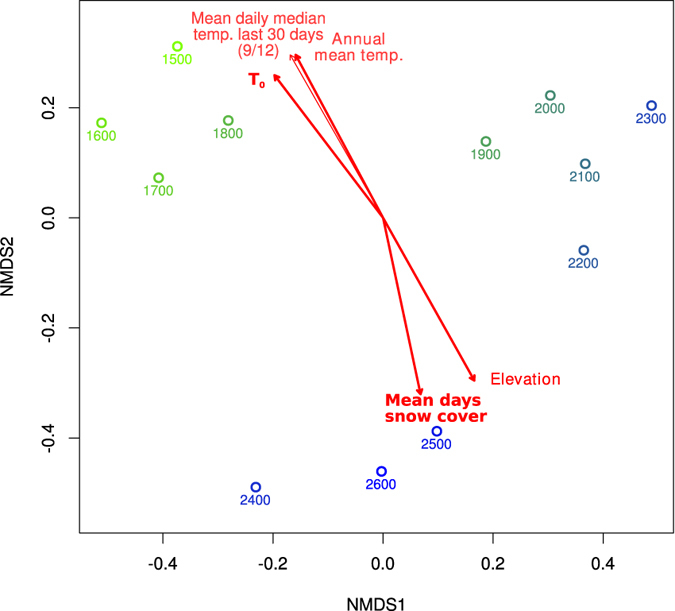
Non-metric multidimensional scaling (NMDS) based on Bray-Curtis dissimilarities of plant community composition. Sites are labelled with elevation in metres and red vectors indicate fitted environmental parameters significantly correlated to NMDS coordinates.

NMDS based on plant composition (Supplementary Table S6) resulted in three clear clusters of samples (Fig. 5), roughly corresponding to the overlapping vegetation zones observed (Table 1), except the subalpine site at 1800 m clustering with montane zone sites. Elevation and associated parameters (soil temperature and days under snow cover) correlated significantly with plant community NMDS coordinates (Fig. 5). Plant composition showed no significant correlation to neither prokaryotic, total eukaryotic or fungal communities (Mantel p > 0.3). Neither did plant richness to soil microbial diversity estimates, nor to other parameters.

### Influence of weather and temporal variation

Compared to annual mean soil temperature, the mean temperature during the last 30 days before sampling correlated more strongly to the prokaryotic community NMDS coordinates (see Supplementary Table S3). To compensate for missing temperature data, elevation was also fitted to the NMDS coordinates where such data was available, resulting in no significant correlation (R^2^ = 0.05, p = 0.4). This may indicate a degree of temporal instability in prokaryotic composition, with relatively recent weather patterns influencing the soil microbial communities significantly. However, according to MANOVA or MRM, neither recent nor annual mean temperatures contributed significantly to explain community dissimilarities in the subset of samples where all such data was available.

## Discussion

Several different elevational patterns of microbial OTU richness have been described, including increasing^15^, decreasing^7^,^8^, hump-backed^13^ and hollow trends^14^. However, such patterns may be better explained by plant community structure, or parameters such as soil pH, C/N, humidity or nutrients co-varying with elevation. The gradient studied here was selected in order to minimise such covariance, with the result that no clear trend in soil microbial richness could be identified. This is consistent with a previous study where an elevation gradient was selected in a similar manner^21^. As opposed to elevation, OTU richness along the gradient studied here correlated positively with C/N. Such correlations have also been described previously^11^, as well as negative ones^10^. Further, soil humidity correlated with the functional diversity of heterotrophic bacteria (NUS), as indicated by Biolog EcoPlates (Table 3 and Fig. 2).

As opposed to alpha-diversity, elevation appeared to influence the composition-based dissimilarity (beta-diversity) of prokaryotic and fungal communities. It thus provided an acceptable proxy for climatic parameters, some of which were also measured. Of these, annual averages of daily maximum soil temperature explained community structure best (Table 4). This influence appeared to be independent of the physicochemical parameters C/N and pH (Table 4). Regardless, C/N appeared to affect prokaryotes and protists more strongly than direct climatic parameters, whereas results based on 18S and ITS amplicons disagreed about the relative strengths of the influences of C/N vs. climate on fungal communities (Table S4). An impressive number of bacterial taxa, nearly one out of four, also correlated directly to C/N (Supplementary Table S5). Thus, C/N appeared as the major driver of prokaryotic and protist community structure along the studied gradient.

Soil pH has often been singled out as the most important influence on soil bacterial communities in studies of elevational gradients^8^,^10^,^14^ and habitats similar to ours^39^,^40^. It also appears to be a key influence on fungal^17^,^20^ and microeukaryote^16^ composition. In the gradient studied here, pH also appeared to influence prokaryotic composition, although not as strongly as C/N. Similar results were obtained in a recent study of arctic tundra soils, when excluding samples from wet sedge^41^. Curiously, neither C nor N concentration in itself appeared to influence community structure, as opposed to previous studies^11^,^41^.

It is difficult to put the influence of the C/N ratio in ecological context without better knowledge of the soil ecosystem dynamics. Carbon and nitrogen availability is associated with the complex interactions between climate, plants, meso- and macrofauna and microbial communities, and involve the ecologically linked processes of litter provision and decomposition, and nitrogen cycling^42^,^43^. It is also probable that grazing contributed to decrease C/N^44^. In the present study, measured C/N ratios were consistently below 20, traditionally interpreted as a net mineralisation of nitrogen^45^. However, soils with lower C/N may also be nitrogen limited^45^, which is supported by the relatively high abundances of Rhizobiales throughout the gradient, indicating an important role for N_2_-fixation. Curiously, relative abundance of Rhizobiales correlated negatively with C/N, indicating higher nitrogen availability where the putative N_2_-fixing rhizobia were more abundant. Thus, other factors than feedback inhibition from nitrate or ammonium were likely most relevant for controlling N_2_-fixation in the studied ecosystem^46^.

Prokaryotic community assembly was also influenced by parent rock (siliclastic vs. carbonate), and orientation (facing south vs. SSW). However, no indicator taxa could be identified for either factor, and differences in parent rock were confounded by pH differences. Sunlight intensity associated with orientation may have affected belowground communities directly or via plants. South-facing sites were also relatively warmer (p < 0.001 using a linear model compensating for elevation), thus indirectly influencing prokaryotic and fungal communities as demonstrated by the influence of average annual soil temperatures on community structure.

Water availability is important for ecosystem functioning. Therefore it is somewhat surprising that soil humidity only correlated to NUS, as opposed to community structure. Biolog profiling has been suggested to mainly reflect functional diversity of currently dominant heterotrophic taxa, as opposed to rare and dormant organisms^47^, perhaps making it a more sensitive indicator to transient changes in community structure due to weather related shifts in soil humidity.

Eukaryotic communities, particularly metazoa, appeared less correlated to environmental parameters than prokaryotes. This can probably be explained by the larger heterogeneity in the composition of these communities (Fig. 4). This may be an effect of the micro-scale patchiness one would expect from larger organisms compared to prokaryotic cells. It is possible that more accurate community structure estimates can be obtained by increasing the amount of soil used for DNA extraction (here 0.25 g), or by replication.

In addition to elevation, it is possible that spatial distance in itself contributed to the observed differences in microbial communities. The existence of such spatial diversity patterns independent of climate or environmental parameters has been indicated previously, even at similar spatial scale^47^, adding to a growing evidence that life history and dispersal is relevant also to microbial biogeography^48^. However, this cannot be assessed using the data presented here, since we used a single gradient where spatial distance was dependent on elevation.

Both richness and composition appeared to correlate between the prokaryotic and eukaryotic communities, particularly fungi with prokaryotes as well as with protists (Table 3, Fig. 3). Similar responses to environmental parameters may have contributed to this. However, it is also likely that part of the explanation is related to synergistic, trophic or competitive interactions between members of these communities. Bacteria-fungal interactions are known to play important roles in the soil ecosystem^49^ and strong correlation between these communities has been observed previously in similar environments^40^,^50^. Examples of possible interactions among the taxa encountered here are the oomycete *Pythium* infecting free-living nematodes^37^ and apicomplexan *Monocystis* infecting annelids. We also expect that bacterial ecto- and endosymbionts of metazoans contributed to the correlation between eukaryotic and prokaryotic communities.

Relatively high abundances of Oomycetes have previously been encountered in a study of grassland soils using a metatranscriptomic approach, thus not subjected to primer and other amplification biases described by Geisen *et al*.^51^. However, the mentioned study encountered relatively low abundances of Apicomplexa compared to ours. As opposed to transcribed rRNA, we also expect to have targeted oocysts^51^, possibly originating from grazing vertebrates infected by cryptosporidiosis, consistent with the classification of the majority of Apicomplexa encountered (although *Monocystis* was also present).

Overall community structure at higher taxonomic ranks was similar to that encountered by Geisen *et al*.^51^, with Rhizaria, followed by Alveolata and Stramenopiles, dominating. Rhizaria was dominated by Cercomonadida followed by Silicofilosea consistent with the same study, as well as low abundance (in average 0.1%) of the typical marine group Choanoflagellida in all samples studied. As expected due to primer mismatch and other issues associated with targeting Amoebozoa^51^, abundances of this group were relatively low, although both Tubulinida, Echinamoebidae and Acanthamoebidae were encountered among the 25 most abundant protist taxa (Fig. 4D).

Fungal community structure (Fig. 4B,C) appeared very similar to mountain and valley pastures in Gorbeia Natural Park in the SW Basque Country (Spain), studied using the same ITS profiling protocol^40^. The same seven taxa dominated with conserved ranking overall in both studies, except for Filobasidales, being more abundant in the present study. The high abundance of Archaeorhizomycetes is particularly interesting, considering the recent debate about its global significance^52^,^53^. The data presented here strengthens previous findings suggesting that it is relatively dominant in alpine grasslands^40^ with evidence from both ITS and 18S rRNA amplicons (mean relative abundance 22% and 21% respectively). However, an alternative explanation could be an unusually high ribosomal gene copy number.

In general, ITS and 18S rRNA amplicon profiling of fungal communities resulted in very consistent results. Together with the broad coverage of protist and metazoan taxa, this illustrates the suitability of the “universal” eukaryotic primers used here. However, lower fungal richness encountered targeting 18S rRNA is consistent with previous comparisons of these markers, indicating lower phylogenetic resolution compared to ITS^54^.

It is well known that above- and belowground communities are tightly interlinked, and involve processes such as root exudation, litter deposition, and interactions ranging from mutualistic symbiosis to plant pathogens and root herbivores^42^. Thus, plant species composition is expected to influence microbial community structure and function (as well as the opposite) and such correlations have been observed on a regional scale, including in Alpine meadows and similar ecosystems^41^,^55^,^56^. However, it is not always possible to assess this effect due to covariation between plant composition with elevation or other environmental parameters influencing both vegetation and belowground communities directly^8^,^9^,^10^. Plants also influence parameters such as soil pH and nitrogen availability^50^.

### In this study plant and belowground communities did not appear correlated

According to ANOSIM (Table 5), both prokaryotic and total eukaryotic community structure differed significantly between the years when samples were collected and samples from 2014 appear to group together in NMDS space (Fig. 3). There was also considerable variability in C/N, soil humidity and NUS between the years (Supplementary Figure S6). Considering the importance of C/N, it is likely that these changes were related. We propose that this physicochemical and biological variation was related to weather patterns such as temperature and rainfall. Consistent with this, averages of recent soil temperature (based on daily medians 1–30 days before sampling) appeared to correlate more strongly to NMDS models of prokaryotic community structure, compared to yearly temperature averages, or elevation. However, more data is necessary to properly assess this hypothesis that could not be confirmed with MANOVA or MRM, likely because temperature data was incomplete. It is also interesting to note that for whole-year estimates of soil temperature, using the daily maxima rather than medians appeared to explain prokaryotic community composition better.

Together, these findings illustrate the importance of long-term studies for understanding the influence of climate change on complex and dynamic ecosystems such as mountainous grasslands, where samples are collected during several years. However, it also underscores the value of continuous monitoring of parameters like soil temperature, rainfall and snow cover, coupled with seasonal monitoring of biological communities. In the present study, we collected a small number of such samples: a week before and after gradient samples in 2013, from late autumn in October, and during snowmelt in May. Unfortunately, it was not possible to separate seasonal temporal variation in community structure from the considerable spatial and inter-annual heterogeneity, even between samples taken from the same sampling station (elevation) and time point.

However, many examples exist of dynamic above- belowground interactions, controlling the activity and abundance of microorganisms over seasonal and shorter time-scales, and seasonal variation in soil microbial composition has been confirmed in several studies^6^,^57^,^58^,^59^.

In conclusion, this study illustrates that microbial communities can be used successfully as indicators of potential impacts from climate change, providing complementary ecological insights. However, it also illustrates the limitations of this approach, such as heterogeneity making results challenging to interpret, particularly for fungi, protists and metazoans. This can hopefully be overcome at least in part by larger scale studies and improved experimental design, such as more frequent sample collection, larger number of replicates, increased sequencing effort and quality. However, we are convinced that the ongoing rapid technological development in terms of throughput and automatisation of molecular techniques will continue, overcoming some of these limitations in a near future.

## Additional Information

**Accession codes:** Data has been deposited to the European Nucleotide Archive with study accession PRJEB12716.

**How to cite this article**: Lanzén, A. *et al*. Multi-targeted metagenetic analysis of the influence of climate and environmental parameters on soil microbial communities along an elevational gradient. *Sci. Rep.* **6**, 28257; doi: 10.1038/srep28257 (2016).

## Supplementary Material

Supplementary Information

Supplementary Dataset 1

Supplementary Dataset 2

Supplementary Dataset 3

## Figures and Tables

**Table 1:** Overview of the studied elevational gradient.

Elevation (m.a.s.l.)	Orientation	Slope (°)	Parent rock*	Vegetation type**	Vegetation cover (%)	Zone
1547	SSW	31.1	S	Dense mesophilic pastures (*Euphrasio-Plantaginetum mediae*)	100	Montane
1604	SSW	13.9	S	Boxwood bushes (*Rhamno saxatilis-Buxetum sempervirentis*)	100	Montane
1705	S	22.3	S	Dense *Festuca eskia* grasses (*Carici pseudotristis-Festucetum eskiae*)	100	Monante/Subalpine
1810	S	21.7	C	“	100	Subalpine
1902	SSW	18.1	S	Rocky limestone pasture (Oxytropido pyrenaicae-Festucetum scopariae)	100	Subalpine
2001	S	22.0	C	“	100	Subalpine
2097	S	22.2	S	“	65	Subalpine
2209	SSW	30.1	S	“	65	Subalpine
2312	S	18.6	S	“	50	Subalpine/Alpine
2390	S	11.4	S	*Alchemillo flabellatae-Nardetum strictae*	85	Alpine
2522	SSW	12.1	C	Culminate calcicole pasture (*Oxytropido foucaudii-Elynetum myosuroidis*)	85	Alpine
2596	SSW	33.3	C	“	70	Alpine

^*^S = Siliciclastic, C = Carbonate

^**^According to Benito^60^.

**Table 2:** Sample and analysis overview.

Sampling date	Stations sampled	Spatial replicates	Temp. monitored*	Abiotic soil properties**	Functl. profiling (Biolog)	16S amplicon seq.***	ITS amp. seq.***	18S amp. seq***
2011–08–31	All	None	No	Yes	Yes	No	No	No
2012–08–30	All	4 × 1700 m, 4 × 2400 m	No	Yes	Yes	Yes	Yes (−2)	Yes (−4)
2013–08–19	1700 m	None	Yes†	No	No	Yes	No	Yes
2013–08–25	All	4 × 1700 m, 4 × 2400 m	Yes†	Yes	Yes ^e.r.^	Yes	Yes	Yes
2013–09–06	1700 m	None	Yes†	No	No	Yes	No	Yes
2013–10–27	1700 m, 2400 m	2 × 4	Yes†	Yes	Yes	Yes^e.r.^	No	Yes^e.r.^
2014–05–08	2400 m	None	Yes	No	No	Yes	No	No
2014–09–10	All	15 × 1700 m, 15 × 2400 m	Yes	Yes	Yes ^e.r.^	Yes^e.r.^	No	No

^*^Soil temperature data available from on-site sensors previous to sampling.

^**^Soil texture (sand, silt and clay), temperature during sampling, humidity, pH, organic matter (%), soil organic matter (SOM), total nitrogen (%).

^***^Number of samples or replicates for which sequencing failed indicated in parentheses if applicable.

^†^During 2012–2013 temperatures were not measured at the 1500, 1600 or 2000 m stations due to equipment failure.

e.r. Excluding replicates. 16S amplicon sequencing was however carried out for 7 replicates from 2014 taken at 2400 m.

**Table 3:** Correlations between physicochemical and biological parameters measured (asterisks represent strength of significance after Bonferroni correction).

Response variable	Explanatory variable	D.f.	Adj. R^2^	Kendall τ	Comparison no. (see Methods)
Clay (%)	Elevation	93	0.26	−0.28**	1
Sand (%)	“	93	0.26	0.23*	1
Temp. during sampling (T_0_)	“	37	0.42	−0.54***	1
Snow cover previous winter	“	21	0.87	0.84***	1
Mean daily temp. 0–30 days prior to sampling	“	21	0.30	−0.51*	1
Annual mean temperature	“	10	0.96	−0.91***	1
Mean annual snow cover (days)	“	10	0.82	0.79**	1
NUS (Biolog)	Soil humidity	64	0.78	0.34*	3
Prokaryotic rarefied richness (RS)	C/N ratio	47	0.24	0.38*	3
Prokaryotic RS	Fungal RS (18S)	35	0.51	0.56***	4
“	Fungal RS (ITS)	34	0.43	0.50**	4
“	Eukaryotic RS	35	0.38	0.53***	4
“	Protist share	35	0.30	0.41*	4
Eukaryotic RS	Fungal RS (18S)	37	0.92	0.84***	4
	Fungal RS (ITS)	31	0.75	0.76***	4
“	Protist share	37	0.75	0.76***	4
“	Fungal share	37	0.39	−0.59***	4
“	Protist RS	37	0.34	0.41*	4
Fungal RS (ITS)	Fungal RS (18S)	31	0.82	0.80***	4
“	Fungal share (18S reads)	31	0.61	−0.62***	4
“	Protist share	31	0.58	0.64***	4
Fungal RS (18S)	Fungal share	37	0.54	−0.64***	4
“	Protist share	37	0.70	0.66***	4
“	Protist RS	37	0.29	0.42**	4

**Table 4:** Results from a MANOVA (adonis) and multiple regression on similarity matrix (MRM) model.

Variable	R^2^ (adonis)	R^2^ (MRM)
C/N ratio	0.18***	0.02***
pH	0.06**	0.03***
Daily max. temp.	0.04*	0.009**
Residuals (unexplained variation)	0.72	0.88

Total prokaryotic community Bray-Curtis dissimilarity was modelled as response variable. Asterisks indicate significance strength.

**Table 5:** Results from Analysis of Similarities (ANOSIM) between communities (Bray-Curtis dissimilarity).

Factor	H_0_	R (16S)	R (ITS)	R (18S)	R (plants)
Year effect	2012 = 2013 = 2014	0.47^**^	0.07	0.13^*^	N/A
Parent rock	siliclastic = carbonate	0.17^*^	−0.07	−0.02	−0.13
Orientation	SSE = SE	0.06^*^	0.00	0.00	−0.02

Asterisks indicate significance strength.

## References

[b1] Parmesan C. Ecological and evolutionary responses to recent climate change. Annu. Rev. Ecol. Evol. S. 37, 637–669 (2006).

[b2] Cardinale B. J. . Biodiversity loss and its impact on humanity. Nature 486, 59–67 (2012). 22678280 10.1038/nature11148

[b3] Grytnes J. A. & McCain C. M. Elevational trends in biodiversity in *Encyclopedia of Biodiversity* (ed. Levin S.) 1–8 (Elsevier, 2007).

[b4] Cannone N., Sgorbati S. & Guglielmin M. Unexpected impacts of climate change on alpine vegetation. Front. Ecol. Environ. 5, 360–364 (2007).

[b5] Gottfried M. . Continent–wide response of mountain vegetation to climate change. Nature Clim. Change 2, 111–115 (2012).

[b6] Bardgett R. D., Bowman W. D., Kaufmann R. & Schmidt S. K. A temporal approach to linking aboveground and belowground ecology. Trends Ecol. Evol. 20, 634–641 (2005). 16701447 10.1016/j.tree.2005.08.005

[b7] Bryant J. A. . Microbes on mountainsides: contrasting elevational patterns of bacterial and plant diversity. Proc. Natl Acad. Sci. USA 105, 11505–11511 (2008). 18695215 10.1073/pnas.0801920105 PMC2556412

[b8] Zhang Y. . Soil bacterial diversity patterns and drivers along an elevational gradient on Shennongjia Mountain, China. Microb. Biotechnol. 8, 739–746 (2015). 26032124 10.1111/1751-7915.12288 PMC4476828

[b9] Wang J.-T. . Altitudinal distribution patterns of soil bacterial and archaeal communities along mt. Shegyla on the Tibetan Plateau. Microb. Ecol. 69, 135–145 (2015). 25074792 10.1007/s00248-014-0465-7

[b10] Shen C. . Soil pH drives the spatial distribution of bacterial communities along elevation on Changbai Mountain. Soil Biol Biochem. 57, 204 - 211 (2013).

[b11] Shen C., Ni Y., Liang W., Wang J. & Chu H. Distinct soil bacterial communities along a small–scale elevational gradient in alpine tundra. Front Microbiol. 6, 582 (2015). 26217308 10.3389/fmicb.2015.00582 PMC4493907

[b12] Singh D., Takahashi K., Park J. & Adams J. M. Similarities and Contrasts in the Archaeal Community of Two Japanese Mountains: Mt. Norikura Compared to Mt. Fuji. Microb Ecol. 71, 428–441 (2015). 26424434 10.1007/s00248-015-0681-9

[b13] Fox J. W. The intermediate disturbance hypothesis should be abandoned. Trends Ecol Evol. 28, 86–92 (2013). 22981468 10.1016/j.tree.2012.08.014

[b14] Singh D. . Strong elevational trends in soil bacterial community composition on Mt. Halla, South Korea. Soil Biol. Biochem. 68, 140–149 (2014).

[b15] Singh D., Shi L. & Adams J. M. Bacterial diversity in the mountains of south-west China: climate dominates over soil parameters. J. Microbiol. 51, 439–447 (2013). 23990294 10.1007/s12275-013-2446-9

[b16] Shen C. . Contrasting elevational diversity patterns between eukaryotic soil microbes and plants. Ecology 95, 3190–3202 (2014).

[b17] Coince A. . Leaf and root-associated fungal assemblages do not follow similar elevational diversity patterns. PLoS ONE 9, e100668 (2014). 24971637 10.1371/journal.pone.0100668 PMC4074112

[b18] Miyamoto Y., Nakano T., Hattori M. & Nara K. The mid-domain effect in ectomycorrhizal fungi: range overlap along an elevation gradient on Mount Fuji, Japan. ISME J. 8, 1739–1746 (2014). 24621523 10.1038/ismej.2014.34 PMC4817612

[b19] Bahram M., Põlme S., Kõljalg U., Zarre S. & Tedersoo L. Regional and local patterns of ectomycorrhizal fungal diversity and community structure along an altitudinal gradient in the Hyrcanian forests of northern Iran. New Phytol. 193, 465–473 (2012). 21988714 10.1111/j.1469-8137.2011.03927.x

[b20] Liu L. . Altitudinal distribution patterns of AM fungal assemblages in a Tibetan alpine grassland. FEMS Microbiol. Ecol. 91, ffv078 (2015). 10.1093/femsec/fiv078 26142427

[b21] Fierer N. . Microbes do not follow the elevational diversity patterns of plants and animals. Ecology 92, 797–804 (2011). 21661542 10.1890/10-1170.1

[b22] Segura D. A. Caracterización mediante teledetección del funcionamiento de los ecosistemas ibéricos. Bases para la conservación de la biodiversidad en un escenario de cambio global. PhD thesis. (University of Almería, 2005).

[b23] Teubner I. E., Haimberger L. & Hantel M. Estimating Snow Cover Duration from Ground Temperature, J. Appl. Meteorol. 54, 959–965 (2015).

[b24] Nelson D. & Sommers L. Total carbon, organic carbon and organic matter in Methods of soil analysis. Part 3. Chemical methods (eds Page A. L. .) (SSSA and ASA, 1996).

[b25] Epelde L., Becerril J. M., Hernández–Allica J., Barrutia O. & Garbisu C. Functional diversity as indicator of the recovery of soil health derived from *Thlaspi caerulescens* growth and metal phytoextraction. Appl. Soil Ecol. 39, 299–310 (2008).

[b26] D’Amore R. . A comprehensive benchmarking study of protocols and sequencing platforms for 16S rRNA community profiling. BMC Genomics 17, 55 (2016). 26763898 10.1186/s12864-015-2194-9 PMC4712552

[b27] Øvreås L., Forney L., Daae F. L. & Torsvik V. Distribution of bacterioplankton in meromictic Lake Sælenvannet, as determined by denaturing gradient gel electrophoresis of PCR–amplified gene fragments coding for 16S rRNA. Appl. Environ. Microbiol. 63, 3367–3373 (1997). 9292986 10.1128/aem.63.9.3367-3373.1997 PMC168642

[b28] Hadziavdic K. . Characterization of the 18S rRNA Gene for Designing Universal Eukaryote Specific Primers. PLoS One 9, e87624 (2014). 24516555 10.1371/journal.pone.0087624 PMC3917833

[b29] McGuire K. L. . Digging the New York City Skyline: Soil Fungal Communities in Green Roofs and City Parks. PloS One 8, e58020 (2013). 23469260 10.1371/journal.pone.0058020 PMC3585938

[b30] Edgar R. C. UPARSE: highly accurate OTU sequences from microbial amplicon reads. Nat. Methods 10, 996–998 (2013). 23955772 10.1038/nmeth.2604

[b31] Martin M. Cutadapt removes adapter sequences from high–throughput sequencing reads. EMBnet.journal 17, 10–12 (2011).

[b32] Rognes T. . *VSEARCH (v1.1.3_linux_x86_64). Technical report.* (2015) Available at: https://github.com/torognes/vsearch (Accessed: 7th June 2015).

[b33] Nilsson R. . Improving ITS sequence data for identification of plant pathogenic fungi. Fungal Divers. 67, 11–19 (2014).

[b34] Lanzén A. . CREST–Classification Resources for Environmental Sequence Tags. PLoS ONE 7, e49334 (2012). 23145153 10.1371/journal.pone.0049334 PMC3493522

[b35] Pilloni G., Granitsiotis M. S., Engel M. & Lueders T. Testing the limits of 454 pyrotag sequencing: reproducibility, quantitative assessment and comparison to T-RFLP fingerprinting of aquifer microbes. PLoS One 7, e40467 (2012). 22808168 10.1371/journal.pone.0040467 PMC3395703

[b36] Oksanen J. . *Vegan: community ecology package. Technical report.* (2013) Available at: http://CRAN.R-project.org/package=vegan (Accessed: 5th August 2015).

[b37] Goslee S. C. & Urban D. L. (2007), ‘The ecodist package for dissimilarity-based analysis of ecological data’, Journal of Statistical Software 22, 1–19.

[b38] Tzean S. S. & Estey R. H. Species of *Phytophthora* and *Pythium* as nematode-destroying fungi. J. Nematol. 13, 160–163 (1981). 19300738 PMC2618058

[b39] Nielsen U. N., Osler G. H. R., Campbell C. D., Burslem D. F. R. P. & van der Wal R. The influence of vegetation type, soil properties and precipitation on the composition of soil mite and microbial communities at the landscape scale. J. Biogeogr. 37, 1317–1328 (2010).

[b40] Lanzén A. . The community structures of prokaryotes and fungi in mountain pasture soils are highly correlated and primarily influenced by pH. Front. Microbiol. 6, 1321 (2015). 26640462 10.3389/fmicb.2015.01321 PMC4661322

[b41] Shi Y. . Vegetation-associated impacts on arctic tundra bacterial and microeukaryotic communities. Appl. Environ. Microbiol. 81, 492–501 (2015). 25362064 10.1128/AEM.03229-14 PMC4277566

[b42] Wardle D. A. . Ecological linkages between aboveground and belowground biota. Science 304, 1629–1633 (2004). 15192218 10.1126/science.1094875

[b43] Jackson L. E., Burger M. & Cavagnaro T. R. Roots, nitrogen transformations, and ecosystem services. Annu. Rev. Plant Biol. 59, 341–363 (2008). 18444903 10.1146/annurev.arplant.59.032607.092932

[b44] Oates L. G., Balser T. C. & Jackson R. D. Subhumid pasture soil microbial communities affected by presence of grazing, but not grazing management. Appl. Soil Ecol. 59, 20–28 (2012).

[b45] Bengtsson G., Bengtson P. & Månsson K. F. Gross nitrogen mineralization-, immobilization-, and nitrification rates as a function of soil C/N ratio and microbial activity. Soil Biol. Biochem. 35, 143–154 (2003).

[b46] Hartwig U. A. The regulation of symbiotic N2 fixation: a conceptual model of N feedback from the ecosystem to the gene expression level. Perspect. Plant Ecol. 1, 92–120 (1998).

[b47] Bissett A., Richardson A. E., Baker G., Wakelin S. & Thrall P. H. Life history determines biogeographical patterns of soil bacterial communities over multiple spatial scales. Mol. Ecol. 19, 4315–4327 (2010). 25241408 10.1111/j.1365-294x.2010.04804.x

[b48] Nemergut D. R. . Patterns and processes of microbial community assembly. Microbiol. Mol. Biol. Rev. 77, 342–356 (2013). 24006468 10.1128/MMBR.00051-12 PMC3811611

[b49] Frey-Klett P. . Bacterial-fungal interactions: hyphens between agricultural, clinical, environmental, and food microbiologists. Microbiol. Mol. Biol. Rev. 75, 583–609 (2011). 22126995 10.1128/MMBR.00020-11 PMC3232736

[b50] Singh B. K., Dawson L. A., Macdonald C. A. & Buckland S. M. Impact of biotic and abiotic interaction on soil microbial communities and functions: A field study. Appl Soil Ecol. 41, 239–248 (2009).

[b51] Geisen S. . Metatranscriptomic census of active protists in soils. ISME J. 9, 2178–2190 (2015). 25822483 10.1038/ismej.2015.30 PMC4579471

[b52] Schadt C. W. & Rosling A. Comment on “Global diversity and geography of soil fungi”. Science 348, 1438 (2015). 26113712 10.1126/science.aaa4269

[b53] Tedersoo L. . Response to Comment on “Global diversity and geography of soil fungi”. Science 349, 936 (2015). 26315429 10.1126/science.aaa5594

[b54] Tedersoo L. . Shotgun metagenomes and multiple primer pair-barcode combinations of amplicons reveal biases in metabarcoding analyses of fungi. MycoKeys 10, 1–43 (2015).

[b55] Zinger L. . Contrasting diversity patterns of crenarchaeal, bacterial and fungal soil communities in an alpine landscape. PLoS One 6, e19950 (2011). 21589876 10.1371/journal.pone.0019950 PMC3093402

[b56] Schmidt S. K. . Plant microbe interactions at multiple scales across a high–elevation landscape. Plant Ecol. Divers (2014).

[b57] Schadt C. W., Martin A. P., Lipson D. A. & Schmidt S. K. Seasonal dynamics of previously unknown fungal lineages in tundra soils. Science 301, 1359–1361 (2003). 12958355 10.1126/science.1086940

[b58] Lipson D. A. & Schmidt S. K. Seasonal changes in an alpine soil bacterial community in the Colorado Rocky Mountains. Appl. Environ. Microbiol. 70, 2867–2879 (2004). 15128545 10.1128/AEM.70.5.2867-2879.2004 PMC404381

[b59] Hill R. . Temporal and spatial influences incur reconfiguration of Arctic heathland soil bacterial community structure. Environ. Microbiol. (2015). 10.1111/1462-2920.13017 26259508

[b60] Benito J. L. Catálogo florístico del Parque Nacional de Ordesa y Monte Perdido (Pirineo aragonés) in Monografías de Botánica Ibérica (ed. Benito J. L.), No. 5 (Jolube, 2006).

