# Supplementary material for: Multi-targeted metagenetic analysis of the influence of climate and environmental parameters on soil microbial communities along an elevational gradient
Source: Sci Rep. 2016 Jun 20;6:28257. doi: 10.1038/srep28257 (PMC4913321; doi:10.1038/srep28257)
Supplement: Supplementary Information [file srep28257-s1.doc]

# SUPPLEMENTARY INFORMATION

# Multi-targeted metagenetic analysis of the influence of climate and environmental parameters on soil microbial communities along an elevational gradient

1. *Anders Lanzén, Lur Epelde, Fernando Blanco, Iker Martín, Unai Artetxe, Carlos Garbisu*

**Supplementary Table S1 – All physicochemical measurements including average temperatures and predicted snow coverage**

*(See separate file)*

**Supplementary Table S2 – Total sequence reads and diversity estimates for the three amplicon library designs**

*(See separate file)*

**Supplementary Table S3 – Correlation matrix, listing Kendall's tau coefficients between all measured parameters**

*(See separate file)*

**Supplementary Table S4 – Correlation of physicochemical and biological parameters to NMDS coordinates** (asterisks represent strength of significance and numbers in parentheses number of samples with data available)

| **Parameter / Community** | **Prokaryotes** | **Fungi (ITS)** | **Eukaryotes (18S)** | **Fungi (18S)** | **Metazoa (18S)** | **Protists (18S)** | **Plants** |
| --- | --- | --- | --- | --- | --- | --- | --- |
| Daily median soil temperature (annual mean) | 0.15* (60/60) | 0.16 (37/37) | 0.02 (39/39) | 0.02 | 0.07 | 0.01 | 0.92*** (12/12) |
| Mean days of snow cover | 0.14* (60/60) | 0.13 (37/37) | 0.02 (39/39) | 0.02 | 0.05 | 0.01 | 0.88*** (12/12) |
| Elevation | 0.13* (60/60) | 0.18* (37/37) | 0.02 (39/39) | 0.02 | 0.08 | 0.01 | 0.94*** (12/12) |
| Daily max. soil temp. (an.mean) | 0.12* (60/60) | 0.16 (37/37) | 0.02 (39/39) | 0.02 | 0.07 | 0.01 | 0.91***  (12/12) |
| T_0_ | 0.04 (60/60) | 0.11 (37/37) | 0.02 (39/39) | 0.05 | 0.09 | 0.07 | 0.87*** (12/12) |
| Slope | 0.08 (60/60) | 0.03 (37/37) | 0.04 (39/39) | 0.03 | 0.01 | 0.04 | 0.03 (12/12) |
| C/N | 0.46*** (49/60) | 0.16 (29/37) | 0.15 (29/39) | 0.25* | 0.12 | 0.23* | 0.04 (12/12) |
| pH | 0.17* (49/60) | 0.10 (29/37) | 0.00 (29/39) | 0.08 | 0.06 | 0.08 | 0.34 (12/12) |
| %Sand | 0.05 (49/60) | 0.10 (29/37) | 0.03 (29/39) | 0.04 | 0.09 | 0.06 | 0.22 (12/12) |
| %Silt | 0.08 (49/60) | 0.05 (29/37) | 0.01 (29/39) | 0.00 | 0.03 | 0.01 | 0.09 (12/12) |
| %Clay | 0.09 (49/60) | 0.11 (29/37) | 0.05 (29/39) | 0.07 | 0.10 | 0.08 | 0.24 (12/12) |
| SOM | 0.09 (49/60) | 0.05 (29/37) | 0.00 (29/39) | 0.02 | 0.01 | 0.00 | 0.16 (12/12) |
| N | 0.11 (49/60) | 0.01 (29/37) | 0.01 (29/39) | 0.02 | 0.02 | 0.02 | 0.14 (12/12) |
| Humidity | 0.01 (41/60) | 0.05 (29/37) | 0.10 (29/39) | 0.01 | 0.03 | 0.01 | 0.01 (12/12) |
| Daily median 0 – 30 days prior | 0.25** (37/60) | 0.02 (16/37) | 0.06 (20/39) | 0.01 | 0.11 | 0.17 | 0.90** (9/12) |

**Supplementary Table S5 – Taxa with abundance significantly correlated to climatic and physicochemical parameters measured**

| **Taxon** | **Library** | **Domain** | **Avg. abundance** | **Parameter** | **p** | **Kendall tau** |
| --- | --- | --- | --- | --- | --- | --- |
| Rhizobiales | 16S | Bacteria | 1.1E-01 | C/N | 2.4E-02 | -0.37 |
| Acidobacteriales (Acidobacteria group 1) | 16S | Bacteria | 9.8E-02 | C/N | 7.7E-05 | -0.48 |
| Chthoniobacterales | 16S | Bacteria | 9.3E-02 | C/N | 5.2E-03 | 0.4 |
| Frankiales | 16S | Bacteria | 6.0E-02 | C/N | 1.8E-06 | -0.54 |
| Xanthomonadales | 16S | Bacteria | 4.5E-02 | C/N | 3.7E-03 | -0.41 |
| Acidobacteria group 2 | 16S | Bacteria | 4.4E-02 | C/N | 1.7E-03 | -0.43 |
| Bacillales | 16S | Bacteria | 3.3E-02 | C/N | 4.0E-02 | 0.36 |
| Acidobacteria group 6 | 16S | Bacteria | 2.7E-02 | C/N | 1.1E-03 | 0.43 |
| Acidobacteria group 3 | 16S | Bacteria | 2.7E-02 | C/N | 6.7E-06 | -0.52 |
| Thermoleophilia AKIW543 | 16S | Bacteria | 1.7E-02 | C/N | 7.3E-03 | 0.4 |
| Corynebacteriales | 16S | Bacteria | 1.5E-02 | pH | 6.3E-03 | 0.41 |
| Chloroflexi Subdivision 10 – KD4-96 | 16S | Bacteria | 1.2E-02 | C/N | 1.1E-05 | 0.51 |
| Nitrosomonadales | 16S | Bacteria | 6.9E-03 | C/N | 3.0E-02 | 0.37 |
| Acidobacteria group 4 | 16S | Bacteria | 6.1E-03 | C/N | 4.7E-04 | 0.45 |
| Cyanobacteria WD272 | 16S | Bacteria | 4.5E-03 | C/N | 6.2E-03 | -0.4 |
| Cytophagales | 16S | Bacteria | 3.3E-03 | C/N | 5.2E-03 | 0.4 |
| Flavobacteriales | 16S | Bacteria | 2.9E-03 | C/N | 1.9E-02 | 0.38 |
| Desulfuromonadales | 16S | Bacteria | 1.9E-03 | Slope | 2.5E-02 | -0.36 |
| Acidobacteria group 17 | 16S | Bacteria | 9.2E-04 | C/N | 6.6E-03 | 0.41 |
| Chloroflexi Subdivision 6 – S085 | 16S | Bacteria | 7.8E-04 | C/N | 5.6E-04 | 0.46 |
| Acidobacteria group 13 | 16S | Bacteria | 7.3E-04 | C/N | 3.5E-02 | -0.36 |
| Thermomicrobia JG30-KF-CM45 | 16S | Bacteria | 6.3E-04 | C/N | 2.2E-04 | 0.48 |
| Verrucomicrobiales | 16S | Bacteria | 5.7E-04 | C/N | 6.1E-03 | 0.41 |
| Chloroflexi Subdivision 10 – P2-11E | 16S | Bacteria | 5.3E-04 | pH | 4.2E-02 | -0.37 |
| Betaproteobacteria TRA3-20 | 16S | Bacteria | 5.0E-04 | C/N | 9.9E-03 | 0.4 |
| Acidobacteria group 22 | 16S | Bacteria | 4.6E-04 | C/N | 5.1E-03 | 0.43 |
| Chloroflexi Subdivision 10 – Gitt-GS-136 | 16S | Bacteria | 2.9E-04 | C/N | 3.6E-04 | 0.5 |
| Nitrospinaceae order incertae sedis | 16S | Bacteria | 2.8E-04 | C/N | 1.7E-03 | 0.46 |
| Caldilineales | 16S | Bacteria | 2.6E-04 | C/N | 4.2E-03 | 0.43 |
| Lactobacillales | 16S | Bacteria | 2.5E-04 | C/N | 7.6E-04 | 0.47 |
| Thermoplasmatales | 16S | Bacteria | 1.7E-04 | C/N | 3.8E-02 | -0.39 |
| Desulfurellales | 16S | Bacteria | 1.2E-04 | C/N | 1.7E-02 | 0.42 |
| Gammaproteobacteria clade KI89A | 16S | Bacteria | 1.1E-04 | C/N | 1.6E-02 | 0.42 |
| Acidobacteria group 11 | 16S | Bacteria | 1.0E-04 | C/N | 4.8E-03 | 0.44 |
| Alphaproteobacteria DB1-14 | 16S | Bacteria | 8.7E-05 | C/N | 2.0E-02 | 0.38 |
| Cyanobacteria ML635J-21 | 16S | Bacteria | 8.2E-05 | C/N | 2.6E-03 | 0.43 |
| Thermotogales | 16S | Bacteria | 5.9E-05 | C/N | 1.1E-02 | -0.42 |
| Gemmatimonadetes terrestrial group GeAT425-EubC11 | 16S | Bacteria | 4.2E-05 | C/N | 1.2E-02 | 0.41 |
| Gemmatimonadetes terrestrial group S0134 | 16S | Bacteria | 3.3E-05 | C/N | 1.5E-03 | 0.46 |
| Gemmatimonadetes terrestrial group BD2-11 | 16S | Bacteria | 3.1E-05 | C/N | 4.1E-03 | 0.45 |
| Acidobacteria group AT-s3-28 | 16S | Bacteria | 2.8E-05 | C/N | 2.1E-02 | 0.41 |
| Chloroflexi group AKIW781 | 16S | Bacteria | 2.3E-05 | C/N | 1.4E-02 | 0.41 |
| MVP-88 Elusimicrobia | 16S | Bacteria | 5.1E-06 | C/N | 3.7E-02 | 0.41 |
| Group I.1c Thaumarchaeota | 16S | Archaea | 5.7E-04 | C/N | 7.2E-03 | -0.42 |

**Table S6 – Plant species distribution along the gradient** (presence / absence)

| **Elevation** | **2600** | **2500** | **2400** | **2300** | **2200** | **2100** | **2000** | **1900** | **1800** | **1700** | **1600** | **1500** |
| --- | --- | --- | --- | --- | --- | --- | --- | --- | --- | --- | --- | --- |
| *Achillea millefolium* | 0 | 0 | 0 | 0 | 0 | 0 | 0 | 0 | 1 | 1 | 1 | 1 |
| *Alchemilla alpina* | 0 | 0 | 0 | 0 | 0 | 0 | 0 | 1 | 1 | 1 | 0 | 0 |
| *Anthyllis vulneraria* | 0 | 0 | 0 | 0 | 0 | 0 | 0 | 0 | 0 | 0 | 0 | 1 |
| *Arenaria moehringioides* | 0 | 0 | 0 | 0 | 0 | 0 | 0 | 0 | 0 | 0 | 1 | 1 |
| *Brimeura amethystina* | 0 | 0 | 0 | 0 | 0 | 0 | 0 | 0 | 0 | 0 | 1 | 0 |
| *Buxus sempervirens* | 0 | 0 | 0 | 0 | 0 | 0 | 0 | 0 | 0 | 0 | 1 | 0 |
| *Campanula cochlearifolia* | 0 | 0 | 0 | 0 | 1 | 0 | 0 | 0 | 0 | 0 | 0 | 0 |
| *Campanula scheuchzeri* | 0 | 1 | 1 | 0 | 0 | 0 | 0 | 0 | 0 | 1 | 0 | 0 |
| *Carduus carlinifolius* | 1 | 0 | 0 | 0 | 0 | 1 | 1 | 1 | 1 | 1 | 1 | 0 |
| *Carex caryophyllea* | 0 | 0 | 0 | 0 | 0 | 0 | 0 | 0 | 1 | 0 | 0 | 0 |
| *Carlina acaulis* | 0 | 0 | 0 | 0 | 0 | 0 | 1 | 1 | 1 | 0 | 0 | 0 |
| *Centaurea jacea* | 0 | 0 | 0 | 0 | 0 | 0 | 0 | 0 | 0 | 0 | 0 | 1 |
| *Cirsium acaule* | 1 | 1 | 1 | 0 | 1 | 0 | 0 | 1 | 0 | 0 | 0 | 0 |
| *Erigeron alpinus* | 0 | 0 | 0 | 0 | 0 | 1 | 0 | 0 | 0 | 0 | 0 | 0 |
| *Eryngium bourgatii* | 0 | 0 | 0 | 0 | 0 | 0 | 0 | 1 | 0 | 0 | 0 | 0 |
| *Euphorbia cyparissias* | 0 | 0 | 0 | 0 | 1 | 0 | 0 | 0 | 0 | 0 | 0 | 0 |
| *Euphrasia stricta* | 0 | 0 | 0 | 0 | 0 | 0 | 0 | 0 | 0 | 0 | 1 | 0 |
| *Festuca eskia* | 0 | 0 | 0 | 0 | 0 | 0 | 0 | 0 | 1 | 1 | 0 | 0 |
| *Festuca gautieri* | 0 | 0 | 0 | 1 | 1 | 1 | 1 | 1 | 0 | 0 | 0 | 0 |
| *Galium verum* | 0 | 0 | 1 | 0 | 0 | 0 | 0 | 0 | 1 | 1 | 0 | 1 |
| *Gentiana verna* | 1 | 0 | 0 | 0 | 0 | 0 | 0 | 0 | 0 | 0 | 0 | 0 |
| *Globularia repens* | 0 | 0 | 0 | 0 | 0 | 0 | 1 | 0 | 0 | 0 | 0 | 0 |
| *Hieracium pilosella* | 0 | 0 | 0 | 0 | 1 | 0 | 0 | 0 | 0 | 1 | 0 | 1 |
| *Iris latifolia* | 0 | 0 | 0 | 0 | 0 | 1 | 1 | 1 | 1 | 1 | 1 | 0 |
| *Koeleria vallesiana* | 0 | 0 | 0 | 1 | 1 | 1 | 1 | 1 | 0 | 0 | 0 | 0 |
| *Leontodon hispidu* | 0 | 0 | 0 | 0 | 0 | 0 | 0 | 0 | 0 | 1 | 0 | 0 |
| *Leontodon pyrenaicus* | 1 | 1 | 0 | 0 | 1 | 1 | 1 | 1 | 0 | 0 | 0 | 0 |
| *Lotus corniculatus* | 1 | 1 | 0 | 0 | 0 | 0 | 0 | 0 | 1 | 1 | 1 | 1 |
| *Meum athamanticum* | 0 | 0 | 0 | 0 | 0 | 0 | 0 | 0 | 1 | 0 | 0 | 0 |
| *Myosotis alpestres* | 0 | 0 | 0 | 0 | 0 | 0 | 0 | 0 | 0 | 0 | 0 | 1 |
| *Nardus stricta* | 0 | 0 | 1 | 0 | 0 | 0 | 0 | 0 | 1 | 0 | 0 | 0 |
| *Plantago media* | 0 | 0 | 0 | 0 | 0 | 0 | 0 | 1 | 1 | 0 | 0 | 1 |
| *Poa alpina* | 1 | 1 | 1 | 0 | 0 | 0 | 0 | 0 | 0 | 0 | 0 | 0 |
| *Potentilla erecta* | 0 | 0 | 0 | 0 | 0 | 0 | 0 | 0 | 1 | 1 | 0 | 0 |
| *Potentilla nivalis* | 1 | 0 | 0 | 0 | 0 | 0 | 0 | 0 | 0 | 0 | 0 | 0 |
| *Rhamnus saxatilis* | 0 | 0 | 0 | 0 | 0 | 0 | 0 | 0 | 0 | 0 | 1 | 0 |
| *Rumex acetosa* | 0 | 0 | 0 | 0 | 0 | 0 | 0 | 0 | 0 | 0 | 0 | 1 |
| *Ruscus aculeatus* | 0 | 0 | 0 | 0 | 0 | 0 | 0 | 0 | 0 | 0 | 1 | 0 |
| *Seseli montanum* | 0 | 0 | 0 | 0 | 0 | 0 | 0 | 1 | 0 | 0 | 0 | 0 |
| *Sideritis hyssopifolia* | 0 | 0 | 0 | 0 | 0 | 1 | 1 | 0 | 0 | 0 | 0 | 0 |
| *Taraxacum dissectum* | 0 | 0 | 0 | 0 | 0 | 0 | 0 | 0 | 0 | 1 | 0 | 0 |
| *Taraxacum officinale* | 0 | 0 | 0 | 0 | 0 | 0 | 0 | 0 | 0 | 0 | 0 | 1 |
| *Thymelaea tinctoria* | 0 | 0 | 0 | 1 | 0 | 0 | 0 | 1 | 0 | 0 | 0 | 0 |
| *Thymus serpyllum* | 0 | 0 | 0 | 0 | 1 | 0 | 0 | 0 | 0 | 0 | 0 | 0 |
| *Trifolium alpinum* | 0 | 0 | 1 | 0 | 0 | 0 | 0 | 0 | 1 | 1 | 0 | 0 |
| *Trifolium montanum* | 0 | 1 | 0 | 1 | 1 | 1 | 0 | 0 | 0 | 0 | 0 | 1 |
| *Trifolium pratense* | 0 | 0 | 0 | 0 | 0 | 0 | 0 | 0 | 0 | 0 | 1 | 1 |
| *Trifolium thalii* | 0 | 1 | 0 | 0 | 0 | 0 | 1 | 1 | 0 | 0 | 0 | 0 |
| *Trisetum flavescens* | 0 | 0 | 0 | 0 | 0 | 0 | 0 | 0 | 0 | 0 | 1 | 0 |
| *Veronica chamaedrys* | 0 | 0 | 1 | 0 | 0 | 0 | 0 | 0 | 0 | 0 | 0 | 0 |
| *Vicia pyrenaica* | 0 | 0 | 0 | 0 | 0 | 0 | 0 | 1 | 0 | 1 | 0 | 1 |
| *Richness* | *8* | *9* | *7* | *4* | *9* | *8* | *9* | *15* | *14* | *14* | *11* | *12* |

***Supplementary Figure S1 –*** *
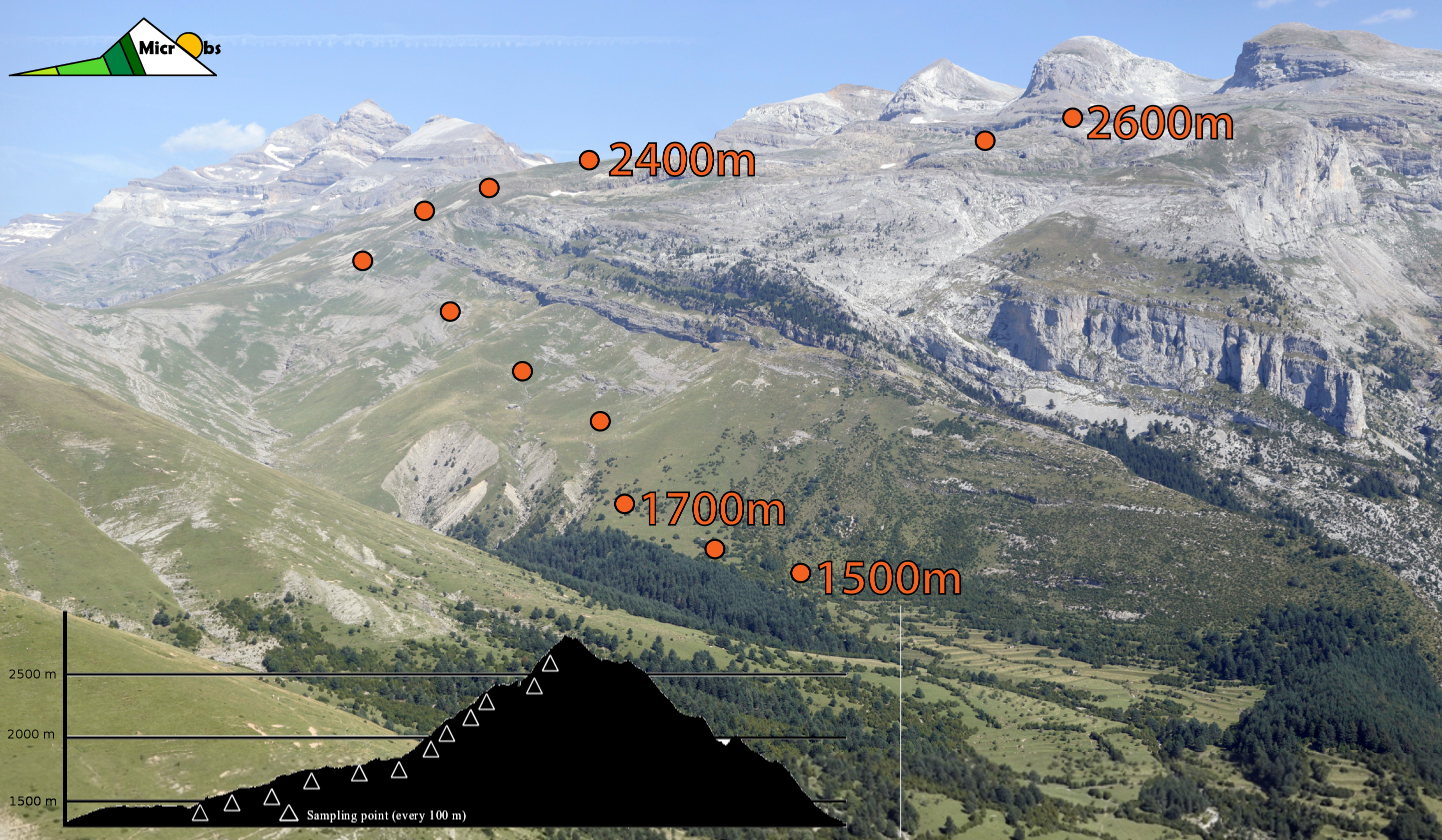
Landscape view and vertical profile of the studied gradient*


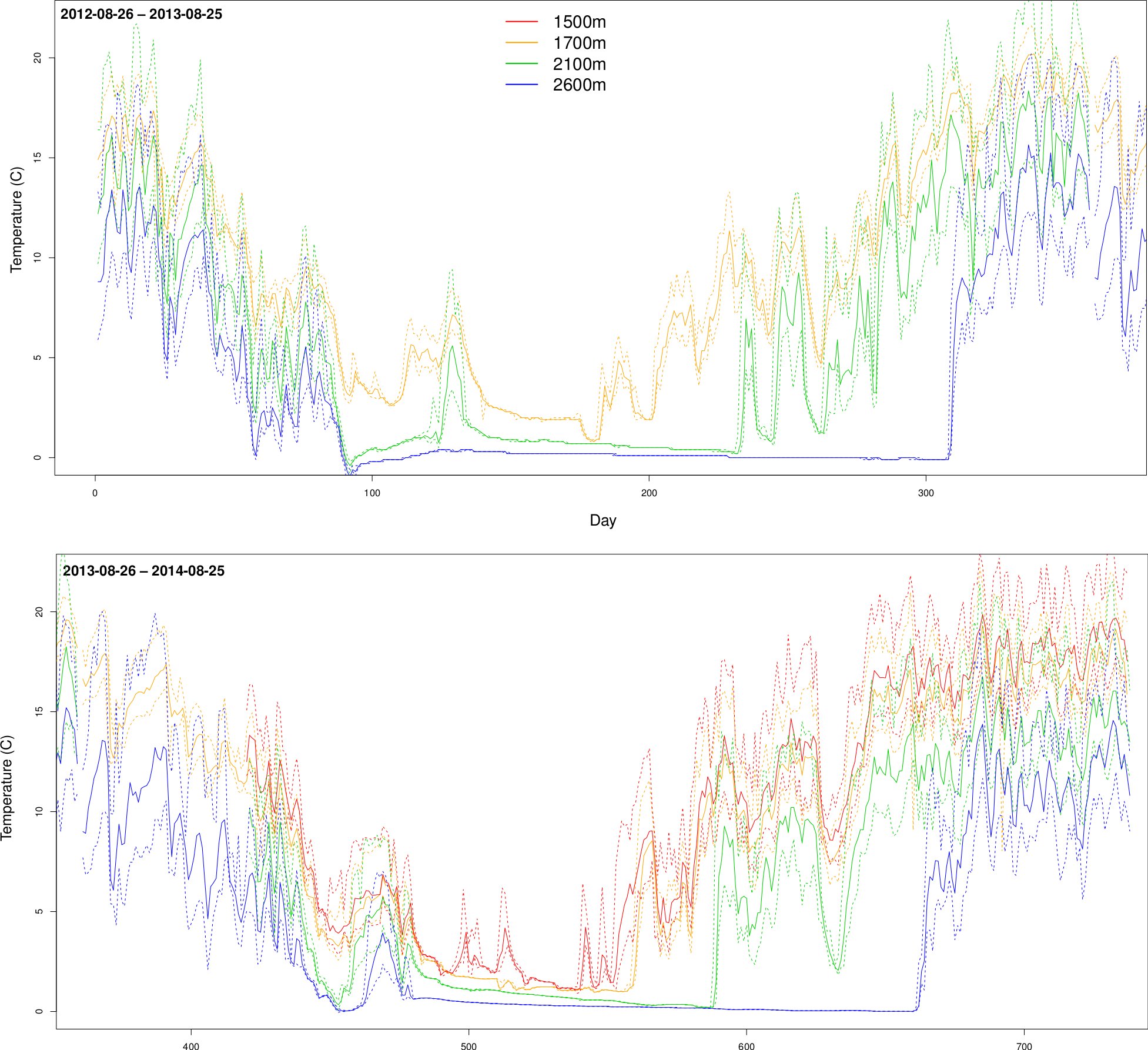


***Supplementary Figure S2 –*** *Daily minimum, median and maximum temperatures as measured by on-site sensors throughout the study for selected sampling stations*


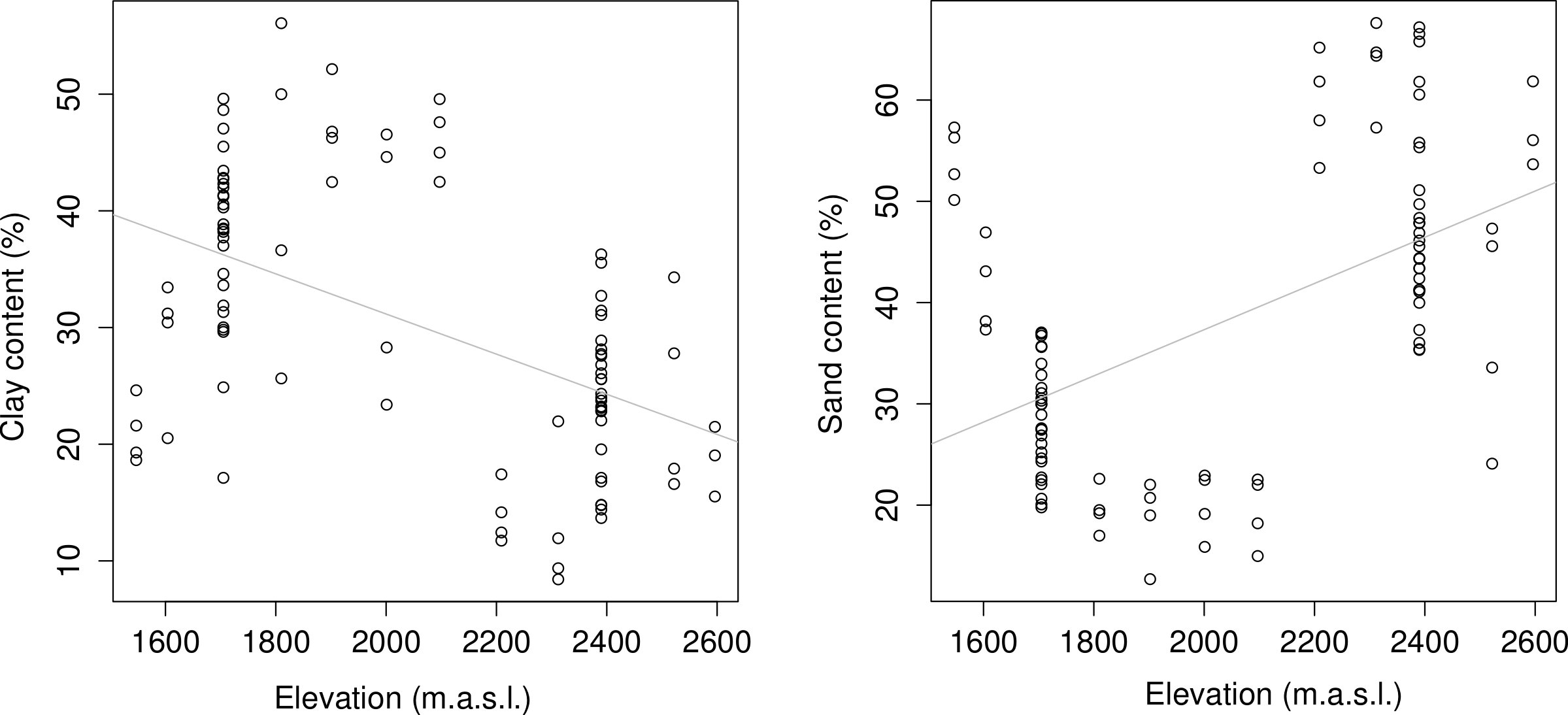


***Supplementary Figure S3 –*** *Soil texture parameters (% sand and clay) with significant rank-correlation to elevation*


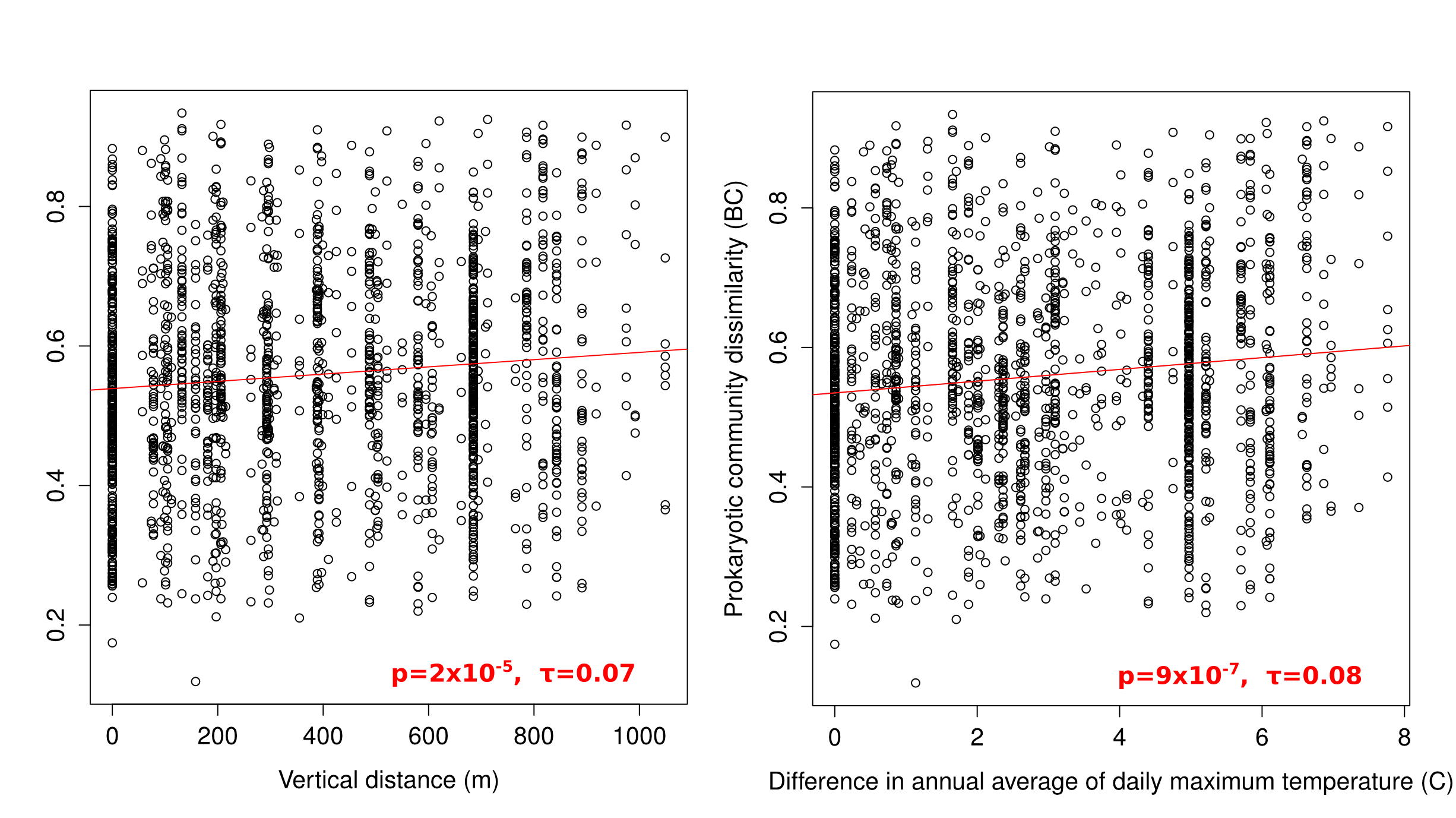


***Supplementary Figure S4 –*** *Pairwise community dissimilarity (Bray-Curtis) plotted vs. elevation difference (left) and difference in annual average of daily maximum temperature (right). Red lines represent best fit according to linear regression.*


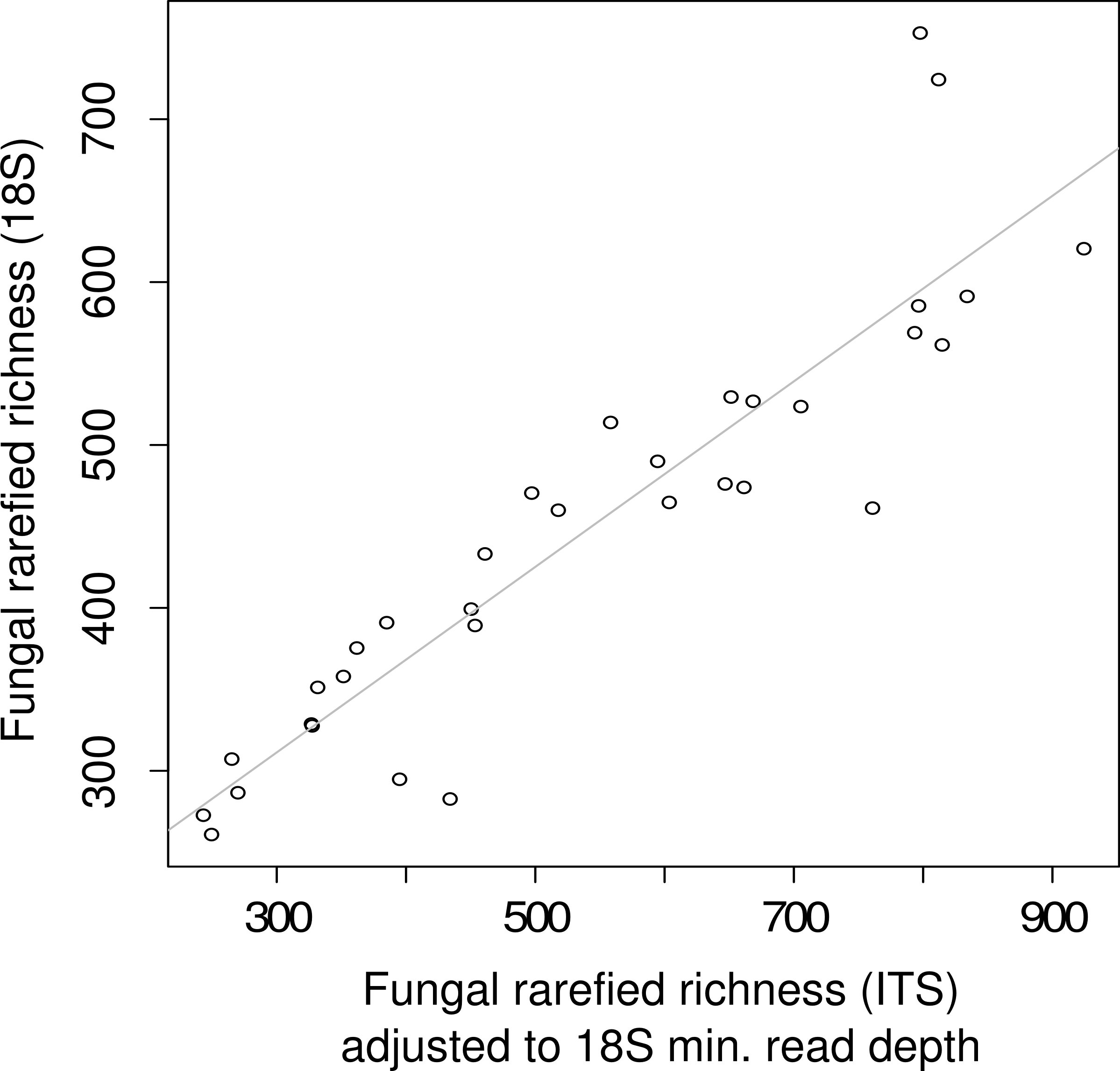


***Supplementary Figure S5 –*** *Fungal rarefied OTU richness in 18S rRNA vs. ITS amplicon datasets. Rarefaction for ITS data was adjusted to minimum read depth in 18S rRNA fungal subsets. The grey line represents a simple linear regression resulting in slope 0.57±0.05 and intercept 140±27*


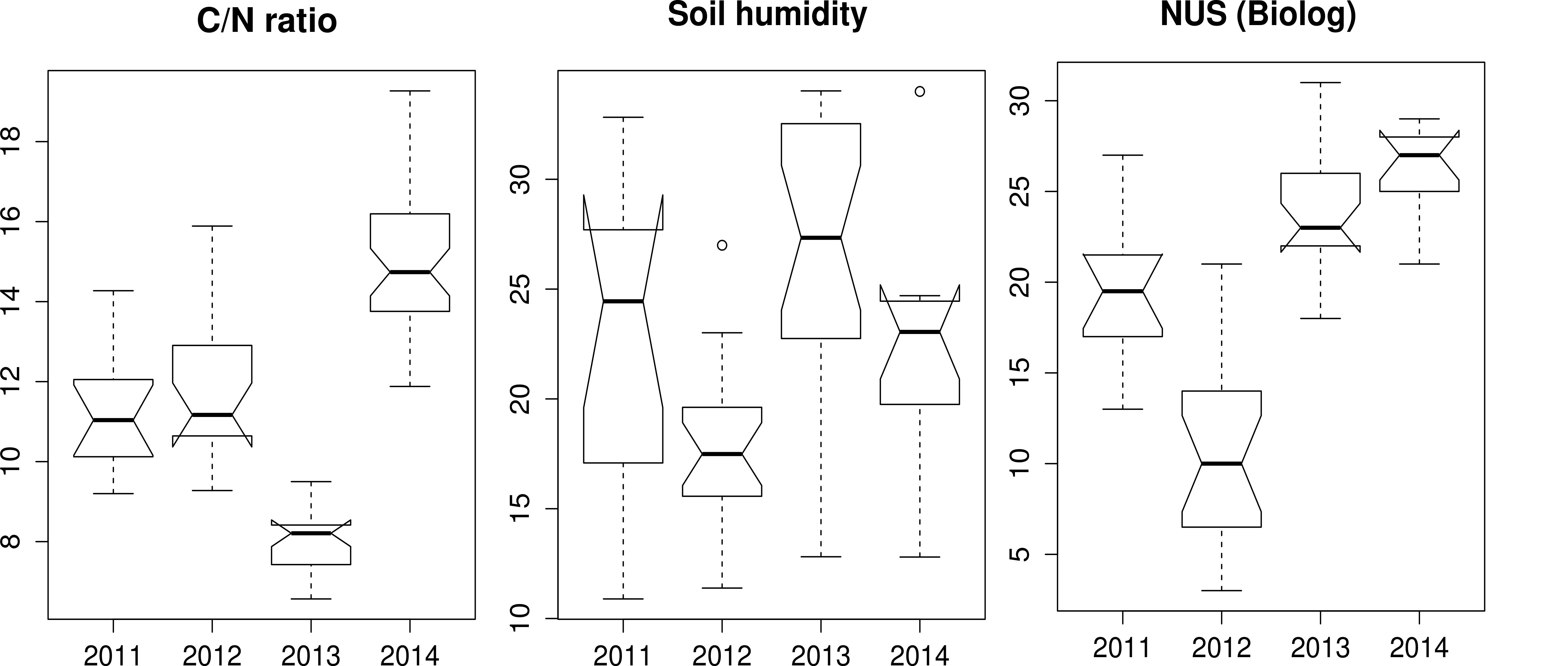


***Figure S6 –*** *Variation in soil C/N ratio, soil humidity and number of utilised substrates (NUS) estimate of heterotrophic functional diversity during the four years of the study*
